# Web-Based Cognitive Bias Modification Program for Young People With Social Anxiety and Hazardous Alcohol Use: Feasibility, Acceptability, and Preliminary Efficacy Study

**DOI:** 10.2196/46008

**Published:** 2023-10-25

**Authors:** Katrina Prior, Elske Salemink, Monique Piggott, Victoria Manning, Reinout W Wiers, Bethany A Teachman, Maree Teesson, Andrew J Baillie, Alison Mahoney, Lauren McLellan, Nicola C Newton, Lexine A Stapinski

**Affiliations:** 1 Matilda Centre for Research in Mental Health and Substance Use The University of Sydney Sydney Australia; 2 Experimental Psychopathology Lab, Department of Clinical Psychology Utrecht University Utrecht Netherlands; 3 Eastern Health Clinical School, Faculty of Medicine, Nursing & Health Sciences Monash University Melbourne Australia; 4 Addiction Development and Psychopathology (ADAPT)-lab, Department of Psychology University of Amsterdam Amsterdam Netherlands; 5 Department of Psychology, School of Arts and Sciences University of Virginia Charlottesville, VA United States; 6 Sydney School of Health Sciences, Faculty of Medicine & Health The University of Sydney Sydney Australia; 7 Clinical Research Unit for Anxiety and Depression, St Vincent's Public Hospital Sydney Australia; 8 School of Psychiatry, University of New South Wales Sydney Australia; 9 Centre for Emotional Health, Department of Psychology Macquarie University Sydney Australia

**Keywords:** alcohol, anxiety, cognitive bias modification, interpretation bias, approach bias, young adult, mobile phone

## Abstract

**Background:**

Interpretation bias modification (IBM) and approach bias modification (ApBM) cognitive retraining interventions can be efficacious adjunctive treatments for improving social anxiety and alcohol use problems. However, previous trials have not examined the combination of these interventions in a young, comorbid sample.

**Objective:**

This study aims to describe the feasibility, acceptability, and preliminary efficacy of a web-based IBM+ApBM program for young adults with social anxiety and hazardous alcohol use (“Re-Train Your Brain”) when delivered in conjunction with treatment as usual (TAU).

**Methods:**

The study involved a 3-arm randomized controlled pilot trial in which treatment-seeking young adults (aged 18-30 y) with co-occurring social anxiety and hazardous alcohol use were randomized to receive (1) the “integrated” *Re-Train Your Brain* program, where each session included *both* IBM and ApBM (50:50 ratio), plus TAU (35/100, 35%); (2) the “alternating” *Re-Train Your Brain* program, where each session focused on IBM or ApBM in an alternating pattern, plus TAU (32/100, 32%); or (3) TAU only (33/100, 33%). Primary outcomes included feasibility and acceptability, and secondary efficacy outcomes included changes in cognitive biases, social anxiety symptoms, and alcohol use. Assessments were conducted at baseline, after the intervention period (6 weeks after baseline), and 12 weeks after baseline.

**Results:**

Both *Re-Train Your Brain* program formats were feasible and acceptable for young adults. When coupled with TAU, both integrated and alternating programs resulted in greater self-reported improvements than TAU only in anxiety interpretation biases (at the 6-week follow-up; Cohen *d*=0.80 and Cohen *d*=0.89) and comorbid interpretation biases (at the 12-week follow-up; Cohen *d*=1.53 and Cohen *d*=1.67). In addition, the alternating group reported larger improvements over the control group in generalized social anxiety symptoms (at the 12-week follow-up; Cohen *d*=0.83) and alcohol cravings (at the 6-week follow-up; Cohen *d*=0.81). There were null effects on all other variables and no differences between the intervention groups in efficacy outcomes.

**Conclusions:**

Should these findings be replicated in a larger randomized controlled trial, *Re-Train Your Brain* has the potential to be a scalable, low-cost, and non–labor-intensive adjunct intervention for targeting interpretation and comorbidity biases as well as generalized anxiety and alcohol-related outcomes in the real world.

**Trial Registration:**

Australian New Zealand Clinical Trials Registry ACTRN12620001273976; https://www.anzctr.org.au/Trial/Registration/TrialReview.aspx?id=364131

**International Registered Report Identifier (IRRID):**

RR2-10.2196/28667

## Introduction

### Background

Social anxiety and alcohol use disorders are 2 substantial public health issues and are among the leading causes of the global burden of disease [1,2]. When they co-occur, there is typically a mutually reinforcing relationship between the disorders that maintains and exacerbates both conditions in a vicious feed-forward cycle (ie, people drink to reduce anxiety in the short term; however, the consequences of drinking, such as shame, guilt, and alcohol withdrawal, lead to greater social anxiety in the long term) [3-5]. This cycle continues and compounds, leading to a greater severity of symptoms, functional impairment, and poorer response to standard treatments [3,6-9].

Both social anxiety and alcohol use disorders are characterized and driven by underlying (modifiable) cognitive biases, but these cognitive mechanisms have largely been examined and treated separately. For instance, some people with social anxiety tend to construe ambiguous stimuli, scenarios, and events in a negative or threatening manner [10-12]. Such “interpretation biases” have been associated with the development, maintenance, and severity of social anxiety symptoms and disorders [13,14]. By contrast, people with alcohol problems often exhibit an automatically triggered tendency to approach (rather than avoid) alcohol [15,16]. This “alcohol approach bias” is implicated in the development of heavy drinking (especially in adolescence and young adulthood), predicts future alcohol use among adults with an alcohol use disorder [16], and has been associated with relapse following treatment [17,18].

There is increasing support for the efficacy of “cognitive bias modification” (CBM) training in reducing anxiety interpretation and alcohol approach biases via the repeated completion of computer-based cognitive tasks [19-25], although there are also many mixed findings [26,27]. Although methodologies vary across studies, “interpretation bias modification” (IBM; predominantly used for anxiety symptoms and disorders) typically involves participants reading a set of ambiguous social scenarios (ie, neither positively nor negatively valanced) and then resolving the ambiguity of the scenarios in a positive or neutral manner. Support for the efficacy of IBM has been garnered by a recent review of meta-analyses [25] and 2 systematic reviews and network meta-analyses [23,24] of CBM programs for anxiety. The reviews concluded that single-session or multisession IBM training among clinical, subclinical, and nonclinical samples can significantly reduce threat-related interpretation biases (and increase positive interpretations) and anxiety levels compared with sham or placebo training or a waitlist control condition. Similar positive findings have been reported in meta-analyses of IBM among children and adolescents specifically (aged <18 y) [28].

Several large randomized controlled trials (RCTs) [29-34] and reviews [19-21] have also provided evidence in support of the efficacy of treatment for alcohol approach biases, known as “approach bias modification” (ApBM), when delivered to clinical samples in conjunction with standard evidence-based treatments, such as cognitive behavioral therapy. In ApBM, individuals are taught to avoid alcohol-related cues by repeatedly making an “avoidance” movement (eg, “pushing away” a joystick or computer mouse) in response to alcohol-related images displayed on a computer screen. Studies show that people who receive 4 to 12 brief ApBM treatment sessions alongside abstinence-oriented residential treatment as usual (TAU) report significantly reduced rates of relapse (by 8%-13%) 1 year later, relative to participants who received TAU only [29-33]. ApBM appears to be particularly beneficial for patients with a comorbid condition, with patients who have a co-occurring anxiety or depressive disorder experiencing a stronger reduction in relapse after ApBM training compared with those with an alcohol use disorder alone [33].

Although these findings are encouraging, empirical support for the efficacy of ApBM programs as stand-alone interventions among nonclinical samples (eg, university students who engage in heavy drinking) is limited, with numerous studies showing null results [20,26,35]. Further, meta-analyses that synthesized findings on the efficacy of these training programs among a mixture of clinical RCTs and nonclinical experimental studies produced similar inconclusive findings (eg, studies by Cristea et al [26] and Cristea et al [27]), perhaps because of substantial heterogeneity in study samples, designs, and methodologies and variations in motivation to change alcohol use [20,36]. Indeed, it has been proposed that successful training effects among participants in clinical RCTs may be due to greater motivation to change their anxiety or alcohol use and that this may be a requisite for successful training effects, as opposed to participants in experimental laboratory studies [20].

To increase the clinical utility and scalability of IBM and ApBM interventions, recent studies have begun investigating the feasibility, acceptability, and efficacy of these programs when delivered via the internet [37-39] or smartphone apps [40,41], rather than in the laboratory or clinic. Both types of technology-based delivery methods have demonstrated considerably good uptake of and adherence to training sessions [40-42]; however, they commonly experience substantial study attrition [38,41,43]. With regard to preliminary efficacy, some *app-based* IBM and ApBM programs have produced significant within-group (from before to after the intervention) reductions in alcohol consumption [41], relapse up to 3 months after treatment [40], cravings and alcohol problem severity 1 month after training [40], and interpretation biases and social anxiety after training compared with a control [44,45]. Other ApBM apps have produced null effects on alcohol use or hazardous drinking [46]. Likewise, *web-based* IBM programs have broadly been efficacious in improving pre-to-post interpretation biases [37,39,42,43,47] and social anxiety symptoms [37,43] (eg, 48% of participants no longer met the criteria for social anxiety disorder after 8 IBM sessions) [37]. Meta-analyses have concluded that web-based IBM programs are efficacious, with no significant difference in efficacy between offline (eg, school or laboratory) and web-based modalities [24]. Although internet-delivered ApBM has been associated with improvements in drinking outcomes among nontreatment-seekers, equivalent reductions have been observed among sham control training groups [38], thus producing nondifferential effects between groups. Future research is needed to integrate web-based ApBM with more traditional cognitive and motivational interventions to improve results [38]. To overcome challenges with attrition and enhance engagement, clinical utility, and intrinsic motivation to complete technology-based CBM training, past research also encouraged the inclusion of a psychoeducational, motivation-enhancement-type module before the first training session [48].

Overall, evidence to date suggests that both IBM and ApBM interventions possess the potential for having positive effects on cognitive biases and social anxiety or alcohol symptoms, particularly among clinical samples. However, it is unknown whether web-based programs are effective among young comorbid samples with clinical symptoms. Given that young people prefer treatments offered via technology (eg, the internet vs face to face) [49] and that the peak onset and disability associated with anxiety and alcohol use disorders occur between adolescence and early adulthood [50,51], the delivery of IBM and ApBM programs via the internet to a clinical sample of young adults may hold particular promise for training effects on anxiety and alcohol use. In addition, a limitation of the existing literature is the predominant focus on IBM and ApBM in isolation of one another. Only 1 small study (N=86) examined the efficacy of CBM programs among people with comorbid anxiety and alcohol use. Specifically, Clerkin and colleagues [52] examined the efficacy of an anxiety-focused and alcohol-focused attention bias CBM program (relative to an anxiety or alcohol sham control) among a sample of adults with comorbid social anxiety and alcohol dependence. The authors found equivalent reductions between the intervention and control conditions in attentional biases, alcohol use disorder, and social anxiety [52]. They concluded that targeting attentional biases alone, without addressing co-occurring biases, may not be a meaningful or clinically significant way to influence comorbid anxiety and alcohol dependence. This aligns with the combined cognitive bias hypothesis, which proposes that biased cognitive processes often act in combination, and “targeting two biases at the same time may enable more rigid cognitive structures to become more malleable” [53].

To better serve the needs of comorbid samples, we developed an internet-delivered IBM+ApBM program for young adults with co-occurring social anxiety and hazardous alcohol use (“Re-Train Your Brain”) and conducted an acceptability study among young adults and clinicians to inform refinements to the intervention [48]. Feedback from both groups indicated that the *Re-Train Your Brain* IBM+ApBM program was an acceptable and potentially clinically useful supplement to TAU [48]; a finding that aligns with previous CBM acceptability studies [54,55]. Despite some valuable feedback on the program, it remains unclear how to incorporate training for both IBM and ApBM. Specifically, it remains unknown whether the format of the *Re-Train Your Brain* program would be most engaging and efficacious if (1) IBM and ApBM tasks are integrated *within* each session (ie, participants receive brief versions of both tasks in a 50:50 ratio) or (2) IBM and ApBM tasks are alternated *between* sessions (ie, participants receive full-length versions of IBM in 1 training session and ApBM in the next training session). The integrated format would provide more repeated practice with smaller gaps between rehearsals of each new skill (which is typically good for learning), but it is possible that the dose may be insufficient on each occasion to have carryover or far transfer effects, thus potentially weakening the effects. It may also be that integrating IBM and ApBM increases engagement because users have more variety in training tasks within each session, thus reducing boredom, which is a common critique of CBM interventions [56,57]. However, regular switching between training tasks may also create fatigue or confusion. Further empirical research is needed to address this research question and determine end user experiences.

### Aims

The aim of this pilot RCT study was to evaluate whether the internet-delivered *Re-Train Your Brain* IBM+ApBM intervention is feasible, acceptable, and preliminarily efficacious as an adjunct to TAU for young adults (aged 18-30 y) who are currently experiencing co-occurring social anxiety disorder symptoms and hazardous alcohol use. As part of this aim, the study assessed whether integrating or alternating the IBM and ApBM training tasks was rated as more acceptable by users and which format demonstrated greater efficacy (primarily based on estimated effect sizes, given that this pilot trial was not powered to detect statistically significant effects).

It was hypothesized that both program formats would be feasible to implement and would be deemed acceptable by young adults. However, it was anticipated that the participants who receive the integrated format would be more engaged and exhibit larger estimated effect sizes, given that the program delivery is more varied *within* each session, potentially reducing the boredom experienced because of repetitive tasks and thus boosting engagement. It was likely that both formats of the *Re-Train Your Brain* program plus TAU would result in greater estimated effect sizes in cognitive biases as well as anxiety- and alcohol-related clinical outcomes over the study period, compared with TAU only.

## Methods

### Trial Design and Registration

**Figure 1:**
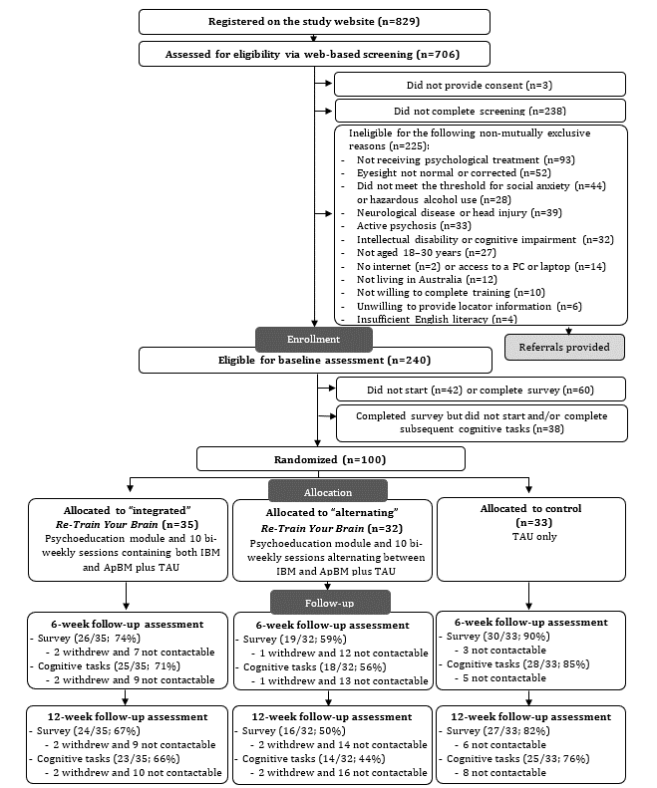
CONSORT (Consolidated Standards of Reporting Trials) diagram showing participant flow through the study. ApBM: approach bias modification; IBM: interpretation bias modification; TAU: treatment as usual.

The *Re-Train Your Brain* study was a 3-arm pilot RCT conducted nationally across Australia. The participants were individually randomized on a 1:1:1 basis to receive (1) an “integrated” *Re-Train Your Brain* program, composed of 10 twice-weekly sessions containing brief versions of both IBM and ApBM (50:50 ratio), plus TAU in accordance with standard practice; (2) an “alternating” *Re-Train Your Brain* program, composed of 10 twice-weekly sessions containing 5 full-length IBM sessions and 5 full-length ApBM sessions, delivered on alternate training days, plus TAU; or (3) TAU only. The assessment end points were 6 weeks and 12 weeks after baseline. Figure 1 depicts the study design.

### Ethical Considerations

The study was prospectively registered with the Australian New Zealand Clinical Trials Registry (ACTRN12620001273976), and ethics approval was granted by the University of Sydney’s Human Research Ethics Committee (#2020/135).

### Participants and Recruitment

#### Overview

Participants were recruited between March 2021 and July 2022 via paid advertising and organic advertising (ie, unpaid, such as posting or sharing of posts) on social media (eg, Facebook [Meta Platforms, Inc], Instagram [Meta Platforms, Inc], and Twitter [Twitter, Inc]), distribution of flyers at educational institutions, and referrals from youth services. On the basis of several rules of thumb used to determine an appropriate sample size for a pilot study [58-60], a sample size of 90 young people (30/arm) was selected to provide sufficient data on the feasibility, acceptability, and preliminary efficacy of the program.

#### Inclusion Criteria

To be eligible, participants were required to (1) be Australian and aged 18 to 30 years; (2) currently report hazardous or harmful alcohol use, as indicated by a score of ≥8 on the Alcohol Use Disorder Identification Test (AUDIT) [61]; (3) currently experience at least mild symptoms of social anxiety, as indicated by a score of ≥7 on the Social Interaction Anxiety Scale or ≥2 on the Social Phobia Scale [62]; (4) have access to the internet via a mouse-operable laptop or PC; (5) receive psychological treatment from a mental health professional (eg, psychologist or counselor) for anxiety, alcohol use problems, or both; and (6) be willing to complete the intervention components.

#### Exclusion Criteria

Owing to possible interference with the participant’s capacity to engage with or understand the cognitive tasks, individuals were excluded if they (1) were unable or unwilling to provide contact details, that is, phone number and email address; (2) self-reported having poor English literacy; (3) had active symptoms of psychosis, as indicated by a score of ≥3 on the Psychosis Screening Questionnaire [63]; (4) had a history of neurological disease or head injury with a loss of consciousness exceeding 30 minutes; (5) had an intellectual disability or cognitive impairment; or (6) had eyesight that was not normal or was corrected to normal (ie, using glasses or contact lenses).

### Measures

The measures are summarized in the subsequent sections and described in detail in the study protocol [64] and Multimedia Appendix 1 [15,32,48,61,62,65-81].

#### Primary Outcome Measures

##### Feasibility

*Feasibility of the research study* was measured through the proportion of participants who provided consent, completed follow-up surveys and/or cognitive assessments, and declined or withdrew from the study. *Feasibility of the intervention* was measured through the proportion of participants who commenced training, completed all 10 training sessions, and completed the optimum number of 6 training sessions for efficacy effects [65]; the number of sessions completed; and the reporting of adverse events.

##### Acceptability

*Usability* of the program was assessed among the intervention groups 6 weeks after baseline using the System Usability Scale [66-68], and *satisfaction* was measured using the 8-item Client Satisfaction Questionnaire [69]. Acceptability was assessed using 13 acceptability items (developed by the research team). To determine which intervention delivery model was preferred, the participants were asked 4 *user experience* items.

#### Secondary Outcome Measures

The following measures were assessed at baseline, after intervention, and 12 weeks after baseline. Multimedia Appendix 1 and the study protocol [64] provide a more detailed description of each measure and its interpretation.

##### Cognitive Biases

*Social anxiety interpretation biases* were measured using the Interpretation Recognition Task [70,71]. Mean similarity ratings for positive target interpretations and negative target interpretations and an overall interpretation bias score were calculated, with higher scores reflecting a stronger interpretation bias [82,83]. *Alcohol approach biases* were assessed using the alcohol Approach Avoidance Task [15]. Scores were calculated separately for alcohol- and non–alcohol-related stimuli, with higher positive values indicating a stronger approach bias for the relevant stimuli. As per previous literature (eg, the study by Piercy et al [72]), to provide an index of alcohol approach bias relative to non–alcohol-related bias, the non–alcohol approach bias scores were then subtracted from the alcohol approach bias scores. *Comorbid interpretation biases for social anxiety and alcohol use* were assessed using the self-report Comorbid Social Anxiety and Alcohol Interpretation Bias task [73]. Higher scores indicate a stronger comorbidity bias.

##### Anxiety and Alcohol Use

*Social anxiety disorder symptoms* were assessed at each time point using the Social Interaction Anxiety Scale and Social Phobia Scale–short forms [62], and generalized social anxiety was measured using the Social Phobia Weekly Summary Scale [74]. *Hazardous alcohol use* was assessed via the AUDIT [61] (score ≥8), whereas *alcohol consumption* (ie, number of drinks per day) in the past month (plus alcohol consumption throughout the intervention period) were assessed via the computerized Timeline Follow-back Procedure [75-77]. *Alcohol dependence* was assessed using the Severity of Alcohol Dependence Questionnaire [78], and *alcohol cravings* was assessed using the Severity of Alcohol Craving Questionnaire–Short Form–Revised [79].

##### Additional Variables

*Sociodemographic characteristics* included age, sex, gender, education, employment status, country of birth, and primary mental health or substance use concern. *Psychological and pharmacological treatments* received in the past 3 months were also ascertained. *Readiness and motivation to change* anxiety and alcohol were assessed via a readiness ruler and the University of Rhode Island Change Assessment [80].

### Intervention Groups: Re-Train Your Brain Intervention Plus TAU

#### Overview

All the participants self-reported that they were currently receiving treatment from a mental health professional for anxiety, alcohol use, or both (ie, TAU). The participants reported predominantly seeing one or more of the following mental health providers: a general practitioner (63/100, 63%), psychologist (49/100, 49%), clinical psychologist (22/100, 22%), or counselor (21/100, 21%).

Both *Re-Train Your Brain* interventions contained an IBM and ApBM component as a supplement to TAU (Multimedia Appendix 1). The participants received access to ten 20-minute training sessions over 5 weeks (approximately 2/wk). Before training, the participants also received immediate access to a web-based psychoeducation and motivational enhancement module, which provided information about anxiety, alcohol use, their interrelationship, the existence of cognitive biases, and the importance of changing these biases. Within the module, the participants set goals for what they hoped to achieve by completing the training. The following is a description of the IBM and ApBM training components.

#### IBM for Social Anxiety

**Figure 2:**
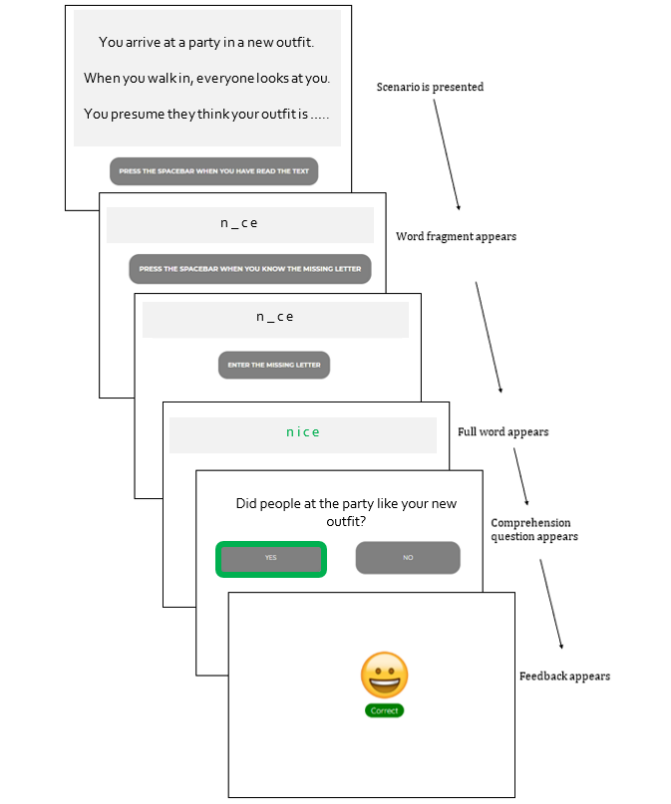
Example interpretation bias modification scenario for illustrating the training procedures.

The participants were provided with a set of ambiguous social scenarios consisting of 3 lines, presented on a computer screen (Figure 2). The final line of each scenario contained a word fragment, and the participants were instructed to fill in the missing letter using their keyboard. The word fragment always resolved the ambiguity in a positive or neutral manner. After each scenario, the participants responded (yes or no) to a comprehension question and received feedback (“correct” or “incorrect,” with a corresponding emoji). Points were awarded for each correct letter and question response. The scenarios were generated specifically for Australian youth [48]. Three practice trials were provided before the real training.

#### ApBM for Alcohol Use

**Figure 3:**
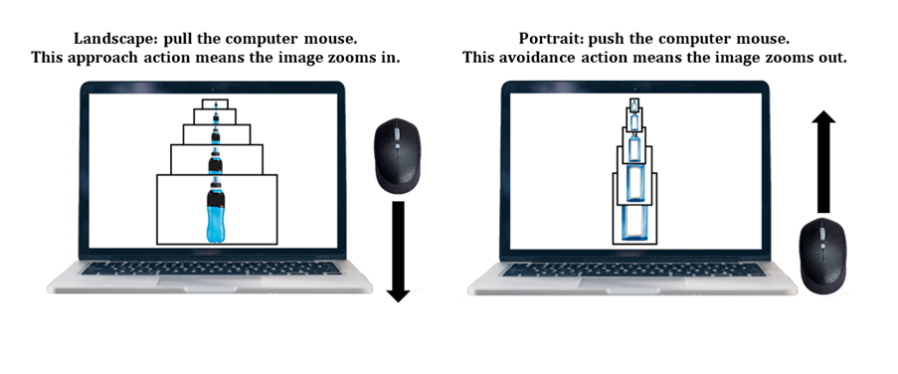
Example approach bias modification scenario for illustrating the training procedures.

The participants were instructed to pull or push a computer mouse in response to the orientation of images containing alcoholic or nonalcoholic beverages (eg, pull landscape and push portrait), and the image size increased or decreased accordingly (Figure 3). The participants received “gamified” feedback for correct (green “✔” plus smiley emoji) and incorrect responses (red “X” plus sad emoji). Points were awarded for each correct movement (+1) to enhance engagement and motivation. The alcoholic and nonalcoholic images were based on beverage types and brands commonly consumed by young Australians [48].

#### Delivery Formats

The *Re-Train Your Brain* intervention was delivered in 2 formats.

### Group 1: “Integrated” Re-Train Your Brain, With Each Twice-Weekly Session Combining Both IBM and ApBM

The participants received 10 training sessions composed of shortened versions of both IBM and ApBM within each session (50:50 ratio, ie, 50% of the duration of each session for IBM and 50% for ApBM, in random order) plus TAU. For IBM training, the participants were presented with 3 blocks of 10 scenarios in each of the 10 sessions (ie, 300 across all sessions). Each block contained 8 positive modification (“induction”) scenarios and 2 filler scenarios, which served 2 functions: first, as most but not all scenarios were positive, there was greater ecological validity, as not all situations resolve positively in daily life; and second, by having some responses that were not positive, the participant could not simply consistently choose positive options for the comprehension questions. Instead, the participants had to read and process each scenario to select the correct answer to the comprehension question. This ensured more elaborate processing of the training material, which would be expected to strengthen the effects of training. The blocks were in a fixed order, but the order of the scenarios within the blocks was random for each participant. For each ApBM training, the participants were presented with 60 portrait or landscape images containing a random selection of 20 alcoholic and 20 nonalcoholic beverages.

### Group 2: “Alternating” Re-Train Your Brain, With Twice-Weekly Sessions Alternating Between IBM and ApBM

The participants in group 2 received the same treatment dose as group 1; however, for each session, the participants received full-length versions of either IBM or ApBM in an alternating fashion (ie, 5 ApBM and 5 IBM sessions, in random order between participants) plus TAU. For IBM training, the participants were presented with 6 blocks of 10 scenarios (ie, 60 scenarios/session; 300 across the 5 IBM sessions). The content of the blocks mirrored that in group 1. For ApBM training, the participants were presented with 120 portrait or landscape images containing 20 alcoholic and 20 nonalcoholic beverages (each image was presented 3 times).

### Control Group (Group 3): TAU Only

The control group received TAU, which included standard care from their existing clinical provider. The participants were placed on a waitlist and were able to access the *Re-Train Your Brain* program after the 12-week follow-up.

### Procedure

Prospective participants were directed to the publicly available *Re-Train Your Brain* website, provided consent, and completed a 10-minute eligibility survey. Eligible participants completed a 30-minute baseline survey, whereas ineligible participants were provided with a list of referral options. The participants who completed the baseline survey were posted a computer mouse for the completion of a baseline cognitive assessment, with email and SMS text message reminders sent to encourage completion.

Immediately following the cognitive assessment, the participants were individually randomized on a 1:1:1 basis to receive (1) the “integrated” *Re-Train Your Brain* intervention plus TAU (35/100, 35%), (2) the “alternating” *Re-Train Your Brain* intervention plus TAU (32/100, 32%), or (3) TAU only (33/100, 33%). Random allocation was performed independently through the trial website using a computer-generated randomization sequence, which was concealed from the investigators. Owing to the nature of the intervention, the participants and research team were not blinded to group allocation. The participants in the intervention groups were given immediate access to the motivational interviewing, psychoeducational module and 2 training sessions per week (every 3-4 d). They were reminded to complete the training via automated emails and SMS text messages. To increase the motivation to complete the training, the participants also received 3 to 4 brief motivational enhancement texts or emails.

At the end of the intervention period (ie, 6 weeks after baseline, allowing for 1 week of flexibility in the rate of training completion) and 12 weeks after baseline, the participants were emailed and asked to complete a web-based follow-up survey and subsequent cognitive assessment. These tasks took 45 to 60 minutes to complete, with an Aus $30 (US $19) e-gift voucher as reimbursement at each time point. To minimize the impact of participant attrition and maximize engagement with the research trial, all the participants were asked to complete 2 weekly 5-minute assessments of their anxiety and alcohol use symptoms, for which they received an Aus $5 (US $3) e-gift voucher per occasion (10 total; maximum Aus $50 [US $32]) at the end of the intervention period.

The intervention and cognitive assessments were accessed via the study website and run using JavaScript, whereas all surveys were delivered via Qualtrics (Qualtrics International Inc). The trial was conducted in accordance with the SPIRIT (Standard Protocol Items: Recommendations for Interventional Trials) 2013 Statement and CONSORT (Consolidated Standards of Reporting Trials) guidelines.

### Statistical Analysis and Power Calculations

Owing to the pilot nature of the study, a formal power calculation was not conducted [84,85]. The current sample was recruited to provide data on the feasibility, acceptability, and preliminary efficacy of the *Re-Train Your Brain* program and determine the expected effect size to inform the sample size of a future RCT. Estimated effect sizes observed in this study should, therefore, be interpreted with some degree of caution.

Descriptive analyses were performed on quantitative feasibility and acceptability outcomes, and linear and logistic regressions were conducted to investigate differences between the intervention groups. For qualitative feedback, a brief general inductive analysis was carried out [86], which first involved a close reading of the data by KP, who identified frequent, dominant, or significant quotes, which were then grouped according to key themes. These themes were then discussed with MP and refined based on discussion and consensus. Direct quotations from the participants have been included to illustrate key themes.

Efficacy analyses were conducted on an intention-to-treat basis using Stata (version 16.0; StataCorp) [87], whereby all participants with complete baseline data were included, regardless of intervention adherence. Multilevel mixed-effects analyses were conducted on continuous dependent variables, and multilevel mixed-effects negative binomial regressions were conducted on count-dependent variables (ie, number of drinks per day). These statistical techniques are rigorous methods for modeling changes over time; they use all the available data and accommodate missing responses using maximum likelihood estimation [88,89]. Normality assumptions were examined, and sensitivity analyses with transformed data were conducted when normality assumptions were violated. All models used baseline measurements as the reference point to estimate participant-specific starting points and change over time. The intervention condition was represented by dummy-coded variables, and a condition by time interaction was examined to assess between-group differences in outcomes over time. The most appropriate model and covariance structure were determined using model fit statistics. The final models included an estimation of random intercept and slope, used an autoregressive error structure for within-person repeated observations over time, and modeled time as a categorical factor. Cohen *d* was calculated from model-estimated β coefficients and SEs to determine the between- and within-group effect sizes at the relevant end points. Effect sizes were interpreted as follows: ≥0.2=small effect, ≥0.5=medium effect, and ≥0.8=large effect [90]. Owing to the preliminary nature of the study, which is not powered for efficacy analyses, the results focus on the magnitude of effect rather than on statistical significance.

## Results

### Sample Characteristics

In total, 100 participants were recruited to the trial; Table 1 lists their sociodemographic and clinical characteristics. The participants were predominantly Australian-born (79/100, 79%) female (76/100, 76%) individuals in their mid-20s (mean 26.44, SD 2.82) who had a Bachelor's degree or higher education (54/100, 54%) and in full-time or part-time employment (72/100, 72%). The participants’ primary concern was most frequently reported to be generalized anxiety (54/100, 54%), with few reporting social anxiety as their main clinical concern (3/100, 3%). However, the majority reported high levels of social anxiety at baseline, with 70% (70/100) meeting both 6-item Social Interaction Anxiety Scale and 6-item Social Phobia Scale criteria for a possible diagnosis of social anxiety disorder. The sample also reported high levels of alcohol use, with a mean past month consumption of 61.61 (SD 71.93) standard drinks and 61% (61/100) reporting AUDIT scores indicative of probable alcohol dependence (scores≥26). It should be noted that although alcohol use was quite high, a sizable proportion (34/100, 34%) reported having 0 drinks in the past month (which appeared to differ between groups: control: 8/33, 24%; integrated intervention: 18/35, 51%; alternating intervention: 8/32, 25%). Over three-quarters (65/100, 65%) of the sample had an interpretation threat bias, whereas 59% (59/100) had an alcohol approach bias (relative to an avoidance bias), with few differences across the 3 groups. Overall, the sample had an equivalent approach bias for alcohol cues relative to nonalcohol cues (mean 1.52, SD 187.72). The participants were predominantly seeing a general practitioner (63/100, 63%) or a psychologist (49/100, 49%) at the time of the baseline survey, and over two-thirds (67/100, 67%) were currently taking medication for anxiety or alcohol use problems. They reported being very ready to change their anxiety (mean 9.07, SD 1.01) and moderately to very ready to change their alcohol use (mean 7.35, SD 2.41).

**Table 1:** Baseline sociodemographic and clinical characteristics, overall and by study group.

			Integrated Re-Train Your Brain + TAU^a^ (n=35)		Alternating Re-Train Your Brain + TAU (n=32)		TAU control (n=33)		Overall (N=100)
Age (y), mean (SD)			26.51 (0.49)		26.47 (2.88)		26.33 (2.72)		26.44 (2.82)
Sex (female), n (%)			27 (77)		22 (69)		27 (82)		76 (76)
**Highest education obtained, n (%)**									
	Secondary school qualification	6 (17)		5 (16)		10 (30)		21 (21)	
	Trade certificate or apprenticeship	5 (14)		2 (6)		3 (9)		10 (10)	
	Other tertiary education	6 (17)		5 (16)		4 (12)		15 (15)	
	Bachelor’s degree or higher	18 (51)		20 (62)		16 (48)		54 (54)	
**Employment status, n (%)**									
	Employed full time	17 (49)		13 (41)		16 (48)		46 (46)	
	Employed part time or casually	11 (31)		9 (28)		6 (18)		26 (26)	
	Full-time home duties	0 (0)		1 (3)		0 (0)		1 (1)	
	Self-employed	1 (3)		1 (3)		1 (3)		3 (3)	
	Full-time student	4 (11)		5 (16)		6 (18)		15 (15)	
	Unemployed	2 (6)		3 (9)		4 (12)		9 (9)	
Australian born, n (%)			28 (80)		25 (78)		26 (79)		79 (79)
**Primary concern (self-reported), n (%)**									
	Generalized anxiety	19 (54)		14 (44)		21 (64)		54 (54)	
	Social anxiety	1 (3)		0 (0)		2 (6)		3 (3)	
	Trauma	4 (11)		3 (9)		2 (6)		9 (9)	
	Depression	5 (14)		8 (25)		7 (21)		20 (20)	
	Alcohol use	4 (11)		5 (16)		1 (3)		10 (10)	
	Other drug use	2 (6)		2 (6)		0 (0)		4 (4)	
Anxiety interpretation bias, n (%)			24 (69)		23 (72)		18 (55)		65 (65)
Alcohol approach bias, n (%)			21 (60)		18 (56)		20 (61)		59 (59)
**Social anxiety severity, n (%)**									
	Possible diagnosis of social anxiety disorder (SIAS-6^b^)	22 (63)		22 (69)		26 (79)		70 (70)	
	Possible diagnosis of social anxiety disorder (SPS-6^c^)	35 (100)		32 (100)		33 (100)		100 (100)	
Total number of drinks in the past month, mean (SD)			49.23 (72.76)		76.88 (81.02)		59.94 (60.15)		61.61 (71.93)
**Hazardous alcohol use severity^d^, n (%)**									
	Medium level	7 (20)		4 (12)		7 (21)		18 (18)	
	High level	10 (29)		5 (16)		6 (18)		21 (21)	
	Probable dependence	18 (51)		23 (72)		20 (61)		61 (61)	
Cannabis use (weekly or greater), n (%)			3 (9)		2 (6)		7 (21)		12 (12)
Other illicit drug use (weekly or greater), n (%)			0 (0)		0 (0)		2 (6)		2 (2)
Nonmedical benzodiazepine use (weekly or greater), n (%)			1 (3)		2 (6)		2 (6)		6 (6)
Readiness to change anxiety, mean (SD)			9.34 (1.14)		9.19 (1.31)		8.67 (1.69)		9.07 (1.01)
Readiness to change alcohol, mean (SD)			8.06 (1.98)		7.25 (2.37)		6.70 (2.72)		7.35 (2.41)
URICA^e^ stages of change, mean (SD)			10.53 (1.48)		9.79 (1.51)		10.11 (1.65)		10.15 (1.57)
**Treatment from a health professional for anxiety or alcohol in past 3 months, n (%)**									
	Counselor	4 (11)		4 (12)		13 (39)		21 (21)	
	Psychologist	24 (69)		15 (47)		10 (30)		49 (49)	
	Clinical psychologist	7 (20)		6 (19)		9 (27)		22 (22)	
	General practitioner	24 (69)		20 (62)		19 (58)		63 (63)	
	Psychiatrist	4 (11)		3 (9)		6 (18)		13 (13)	
	Hospital inpatient admissions	2 (6)		2 (6)		1 (3)		5 (5)	
	Inpatient or residential treatment	1 (3)		0 (0)		1 (3)		2 (2)	
	Emergency department visits	3 (9)		4 (12)		1 (3)		8 (8)	
	Other	1 (3)		0 (0)		3 (9)		4 (4)	
**Medication for anxiety or alcohol in past 3 months**									
	Any medication, n (%)	22 (63)		21 (66)		24 (73)		67 (67)	
	Number of medications, mean (SD)	0.97 (0.99)		1.03 (0.93)		1.24 (1.00)		1.08 (0.97)	

^a^TAU: treatment as usual.

^b^SIAS-6: 6-item Social Interaction Anxiety Scale.

^c^SPS-6: 6-item Social Phobia Scale.

^d^For descriptive purposes, Alcohol Use Disorder Identification Test hazardous alcohol use total scores are grouped here according to categories of increasing alcohol risk, from medium level (scores 8-15) to high level (scores 16-19) to indication for alcohol dependence (scores of ≥20).

^e^URICA: University of Rhode Island Change Assessment Scale.

Despite random allocation to conditions, eyeballing the descriptive statistics revealed several chance differences in baseline characteristics between the groups. Compared with the control group, the integrated *Re-Train Your Brain* group appeared to have lower social anxiety scores, higher readiness to change anxiety, and higher alcohol use scores and were less likely to have seen a counselor and more likely to have seen a psychologist over the past 3 months. They were also more likely to have had 0 drinks in the past month. Compared with the control group, the alternating *Re-Train Your Brain* group appeared to be more likely to have ever used cannabis and less likely to have seen a counselor over the past 3 months. Compared with the alternating *Re-Train Your Brain* group, the integrated *Re-Train Your Brain* group had lower social anxiety scores. As per the CONSORT 2010 guidelines, no tests of the significance of baseline differences between the groups were conducted [91]; however, these baseline differences were accommodated within the analyses, which modeled changes over time from participant-specific starting points estimated using baseline measurements. In response to an honesty screener [92], 99% (99/100) of the participants reported that their survey responses were truthful.

### Primary Outcomes

#### Overview

Feasibility and acceptability outcomes are listed in Table 2 and detailed in the subsequent sections.

**Table 2:** Primary outcomes by group at 6-week and 12-week follow-up assessments.

		Integrated Re-Train Your Brain + TAU^a^ (n=35)	Alternating Re-Train Your Brain + TAU (n=32)	TAU control (n=33)
**Completed follow-up survey, n (%)**				
	6-week follow-up	26 (74)	19 (59)	30 (91)
	12-week follow-up	24 (69)	16 (50)	27 (82)
**Completed follow-up survey and games, n (%)**				
	6-week follow-up	25 (71)	18 (56)	28 (85)
	12-week follow-up	23 (66)	14 (44)	25 (76)
**Study withdrawals, n (%)**				
	6-week follow-up	2 (6)	1 (3)	0 (0)
	12-week follow-up	2 (6)	2 (6)	0 (0)
Completed psychoeducational module, n (%)		27 (77)	24 (75)	N/A^b^
Commenced training, n (%)		28 (80)	26 (81)	N/A
Number of sessions completed, mean (SD)		5.69 (4.28)	5.28 (4.04)	N/A
Completed the optimum of 6 training sessions, n (%)		19 (54)	14 (44)	N/A
Completed all 10 training sessions, n (%)		14 (40)	11 (34)	N/A
Adverse events (at 6-week follow-up), n (%)		4 (11)	0 (0)	5 (15)
Program usability (SUS^c^), mean (SD)		85.40 (11.17)	82.11 (12.97)	N/A
Client satisfaction (CSQ^d^), mean (SD)		24.08 (4.99)	25.63 (5.12)	N/A

^a^TAU: treatment as usual.

^b^N/A: not applicable.

^c^SUS: System Usability Scale.

^d^CSQ: Client Satisfaction Questionnaire.

#### Feasibility

##### Study Feasibility

Figure 1 illustrates the flow of participants through the study phases. Of the 706 patients who were screened for eligibility, 3 (0.4%) did not provide consent, 238 (33.7%) did not complete the screener, and 225 (31.9%) did not meet the inclusion criteria. Of the 240 (34%) eligible participants, 138 (57.5%) completed the baseline survey, of whom 100 (72.5%) then completed the subsequent cognitive assessment and were randomized (ie, 100/240, 41.7% of eligible individuals). Recruitment ceased after randomizing 100 participants. A total of 73 (73%) participants provided survey and cognitive assessment data for at least 1 of the 2 follow-up time points; the 6-week survey was completed by 75% (75/100) of the participants, and 71% (71/100) completed the 6-week cognitive assessment. The rates of completion of both 6-week tasks were significantly different between the control and alternating *Re-Train Your Brain* groups (odds ratio [OR] 0.23, 95% CI 0.07-0.75; *P*=.02) but not between the integrated group and the control or alternating *Re-Train Your Brain* group (OR 0.45, 95% CI 0.13-1.45; *P*=.19; OR 1.94, 95% CI 0.71-5.35; *P*=.20). At the 12-week follow-up, 67% (67/100) of the participants completed the survey, whereas 62% (62/100) of the participants completed both the survey and cognitive assessment. The rate of completion of at least 1 follow-up time point was significantly different between the control (30/33, 91%) and alternating intervention (18/32, 56%; OR 0.01, 95% CI 0.03-0.51; *P*=.004) groups, whereas no significant differences were evident between the integrated group (25/35, 71%) and either the control or alternating group (OR 0.25, 95% CI 0.06-1.01; *P*=.051; OR 1.94, 95% CI 0.71-5.35; *P*=.19). No differences in the baseline sociodemographic or clinical characteristics were identified between those who were lost to follow-up and those who were not. Across the study, of the 100 participants, only 4 (4%) actively withdrew their consent; 3 (3%) withdrew from both follow-up occasions, and 1 (1%) withdrew from the 12-week follow-up only. None of them asked to withdraw their data. No significant differences in withdrawals were observed between the groups (*P*<.05). Overall, these findings suggest that the study was feasible.

##### Intervention Feasibility

Among the 67 participants in the intervention groups, over three-quarters (n=51, 76%) completed the psychoeducational anxiety-alcohol module, and 81% (n=54) completed at least 1 training session, with an average of 5.49 (SD 4.14) sessions completed. No significant differences were found between the integrated and alternating *Re-Train Your Brain* groups in module completion (OR 1.13, 95% CI 0.37-3.46; *P*=.84), training commencement (OR 0.92, 95% CI 0.27-3.11; *P*=.90), or the number of sessions completed (β=.40*,* 95% CI –1.63 to 2.44; *P*=.69). Nearly half (33/67, 49%) of those in the intervention groups completed the optimum dose of 6 training sessions, and 1 in 3 (25/67, 37%) completed all 10 sessions (25/54, 46% of those who completed at least 1 training session). There were no significant differences in the rate of completing 6 training sessions (OR 1.53, 95% CI 0.58-4.01; *P*=.39) or all 10 sessions (OR 1.28, 95% CI 0.47-3.44; *P*=.52) between the integrated and alternating intervention groups.

##### Adverse Events

When asked about adverse events at the 6-week assessment, of the 75 participants who completed the survey, 9 (12%) reported negative or adverse effects during the intervention period (n=4, 44% in the integrated *Re-Train Your Brain* group and n=5, 55% in the control group); however, all these participants explicitly attributed the adverse event to reasons or circumstances external to the *Re-Train Your Brain* program (n=7, 78% attributed this to TAU and n=2, 22% to other reasons). No serious adverse events or adverse events were reported in the open feedback questions or spontaneously reported to the study team.

#### Acceptability

##### Usability

The mean System Usability Scale score across the intervention groups was 83.98 (SD 11.95), which was the highest possible rating and equivalent to an “A” grade. There were no significant differences in the System Usability Scale scores based on delivery modality (ie, integrated vs alternating; β=3.30, 95% CI –4.06 to 10.65; *P*=.37).

##### Satisfaction

The mean 8-item Client Satisfaction Questionnaire score was moderate to high at 24.75 (SD 5.05), with no significant differences in scores between the integrated and alternating *Re-Train Your Brain* groups (β=−1.55, 95% CI –4.65 to 1.55; *P*=.32).

##### Acceptability

The vast majority of the 67 participants in the intervention groups indicated that the program they completed was acceptable (n=59, 88%), user-friendly (n=61, 91%), credible (n=58, 87%), simple (n=64, 96%), and easy to complete (n=61, 91%). Three-quarters (50/67, 75%) said that they could see the potential value and effectiveness of the intervention. Conversely, fewer participants deemed the program to be enjoyable (37/67, 55%), motivating (29/67, 43%), logical (43/67, 64%), engaging (26/67, 39%), likely to be effective in reducing anxiety and alcohol use (38/67, 57%), and helpful in practicing the skills learned in TAU (35/67, 52%). Half (34/67, 51%) of the participants also reported that the training tasks took up too much time. No significant differences were found between the integrated and alternating groups on 3 of the 4 user experience items, including motivation to train (11/35, 31% vs 11/32, 34%; OR 0.87*,* 95% CI 0.56-1.35; *P*=.53), training enjoyment (13/35, 37% vs 9/32, 28%; OR 1.36*,* 95% CI 0.87-2.12; *P*=.17), and delivery of training (17/35, 49% vs 16/32, 50%; OR 0.96*,* 95% CI 0.64-1.45; *P*=.85). However, the integrated group was less likely than the alternating group to rate their training as “very or extremely” simple and user-friendly (26/35, 74% vs 29/32, 91%; OR 0.37*,* 95% CI 0.21-0.67; *P*<.001).

##### Most and Least Helpful Components

The ApBM training was often reported to be fun, especially when trying to complete the tasks quickly:

The picture game was a good challenge to do as fast as possible.

#3637

The IBM training, by contrast, was commonly reported as the most helpful component of the intervention and had practical implications:

The anxiety training gave me a new perspective in viewing my thoughts.

#1260

I could tell from the anxiety section of the program that it was training your brain to consider all options as opposed to only catastrophic ones...I actually have started practicing addressing any anxious thoughts I have about how I am perceived by others.

#1145

The participants also indicated that they found the twice-weekly check-in assessments of their anxiety and alcohol consumption useful for accountability and to reflect on:

Having to interrogate and track my drinking habits and calculate how many drinks I’d consumed over the past week.

#4717

The team also received unsolicited feedback (via text) indicating the impact of the *Re-Train Your Brain* intervention:

I’ve been very grateful to be involved in this study...[the training] has supported me in beginning to limit my alcohol intake and even take on the idea of going sober completely!

#4801

### Secondary Outcomes

#### Overview

Table 3 provides descriptive statistics of the cognitive and clinical variables, and Tables 4 and 5 summarize the efficacy findings and their associated effect sizes.

**Table 3:** Secondary outcomes by group at baseline and 6-week and 12-week follow-up assessments.

			Integrated Re-Train Your Brain + TAU^a^ (n=35), mean (SD)		Alternating Re-Train Your Brain + TAU (n=32), mean (SD)		TAU control (n=33), mean (SD)
**Anxiety interpretation bias (IREC-T^b^)**							
	Baseline	0.24 (0.64)		0.31 (0.61)		0.21 (0.77)	
	6-week follow-up	−0.75 (0.79)		−0.75 (0.76)		0.25 (0.77)	
	12-week follow-up	−0.20 (0.25)		−0.19 (0.47)		−0.11 (0.27)	
**Positive target interpretation (IREC-T)**							
	Baseline	2.42 (0.40)		2.44 (0.50)		2.48 (0.50)	
	6-week follow-up	2.82 (0.50)		2.88 (0.48)		2.32 (0.48)	
	12-week follow-up	2.86 (0.43)		2.94 (0.37)		2.51 (0.46)	
**Negative target interpretation (IREC-T)**							
	Baseline	2.66 (0.54)		2.75 (0.51)		2.69 (0.70)	
	6-week follow-up	2.07 (0.52)		2.13 (0.42)		2.57 (0.71)	
	12-week follow-up	2.66 (0.36)		2.75 (0.43)		2.40 (0.45)	
**Alcohol approach bias (AAT^c^)**							
	Baseline	24.96 (121.42)		14.13 (193.16)		48.26 (138.89)	
	6-week follow-up	−4.26 (101.83)		9.75 (82.07)		33.61 (137.60)	
	12-week follow-up	34.89 (98.61)		−16.23 (131.29)		−7.48 (164.56)	
**Comorbid social anxiety and alcohol interpretation bias**							
	Baseline	3.07 (1.48)		3.31 (1.40)		2.92 (1.46)	
	6-week follow-up	2.47 (1.50)		2.68 (1.52)		2.53 (1.42)	
	12-week follow-up	2.00 (1.69)		2.18 (1.31)		2.68 (1.49)	
**Social anxiety (SIAS-6^d^ and SPS-6^e^)**							
	Baseline	17.91 (8.21)		22.88 (10.76)		23.12 (11.17)	
	6-week follow-up	17.73 (9.85)		19.68 (10.39)		20.87 (10.46)	
	12-week follow-up	15.38 (8.26)		18.44 (8.20)		22.07 (9.66)	
**Generalized social anxiety (SPWSS^f^)**							
	Baseline	4.45 (1.39)		4.92 (1.47)		4.88 (1.39)	
	6-week follow-up	3.98 (1.65)		3.73 (1.93)		4.34 (1.66)	
	12-week follow-up	3.48 (1.39)		2.91 (1.84)		4.24 (1.78)	
**Number of drinks per day in the past month (TLFB^g^)**							
	Baseline	1.64 (2.43)		2.56 (2.70)		2.00 (2.00)	
	6-week follow-up	2.52 (2.26)		2.70 (2.65)		2.50 (2.19)	
	12-week follow-up	3.07 (4.22)		2.15 (1.94)		2.34 (2.19)	
**Hazardous alcohol use (AUDIT^h^)**							
	Baseline	21.46 (6.49)		22.94 (6.56)		21.33 (5.99)	
	6-week follow-up	17.73 (8.39)		17.63 (9.48)		17.47 (6.73)	
	12-week follow-up	15.54 (9.38)		14.19 (8.80)		17.37 (7.76)	
**Alcohol craving (ACQ^i^)**							
	Baseline	3.59 (1.07)		3.53 (1.29)		3.55 (1.14)	
	6-week follow-up	2.97 (1.12)		2.35 (1.36)		3.34 (1.34)	
	12-week follow-up	2.31 (1.02)		2.33 (0.49)		2.73 (0.90)	
**Alcohol dependence (SADQ^j^)**							
	Baseline	19.49 (10.61)		19.06 (9.13)		18.42 (12.36)	
	6-week follow-up	16.42 (13.31)		13.53 (10.55)		15.33 (11.31)	
	12-week follow-up	14.21 (10.73)		12.38 (9.71)		17.04 (12.84)	

^a^TAU: treatment as usual.

^b^IREC-T: Interpretation Recognition Task.

^c^AAT: Alcohol Approach Task.

^d^SIAS-6: 6-item Social Interaction Anxiety Scale.

^e^SPS-6: 6-item Social Phobia Scale.

^f^SPWSS: Social Phobia Weekly Summary Scale.

^g^TLFB: Timeline Follow-back Procedure.

^h^AUDIT: Alcohol Use Disorder Identification Test.

^i^ACQ: Severity of Alcohol Craving Questionnaire–Short Form–Revised.

^j^SADQ: Severity of Alcohol Dependence Questionnaire.

**Table 4:** Mixed models for repeated measures fitted to cognitive bias outcomes with time period and group^a^.

Outcomes				Baseline to 6-week follow-up							Baseline to 12-week follow-up				
				β (95% CI)		Cohen *d*		*P* value		β (95% CI)			Cohen *d*		*P* value
**Anxiety interpretation bias (IREC-T^b^)**															
	**Time effect**														
		Integrated Re-Train	−.99 (−1.29 to −.70)		1.48		<.001		−.44 (−.77 to −0.11)			0.66		.01	
		Alternating Re-Train	−1.08 (−1.42 to −.75)		1.61		<.001		−.51 (−.90 to −.13)			0.76		.01	
		Control	.04 (−.25 to .32)		0.05		.81		−.30 (−.63 to .03)			0.44		.08	
	**Group × time interaction**														
		Integrated Re-Train vs control	−1.03 (−1.44 to −.62)		1.53		<.001		−.14 (−.61 to .32)			0.21		.55	
		Alternating Re-Train vs control	−1.12 (−1.56 to −.68)		1.67		<.001		−.21 (−.72 to .29)			0.32		.40	
		Integrated Re-Train vs alternating Re-Train	.09 (−.36 to .54)		0.14		.69		.07 (−.43 to .58)			0.11		.78	
**Positive target interpretation (IREC-T)**															
	**Time effect**														
		Integrated Re-Train	.40 (.23 to .58)		0.87		<.001		.45 (.25 to .64)			0.97		<.001	
		Alternating Re-Train	.46 (.25 to .66)		0.99		<.001		.54 (.31 to .78)			1.18		<.001	
		Control	−.14 (−.31 to .02)		0.31		.09		.01 (−.18 to .20)			0.02		.91	
	**Group** × **time interaction**														
		Integrated Re-Train vs control	.54 (.30 to.79)		1.18		<.001		.44 (.16 to .71)			0.94		.002	
		Alternating Re-Train vs control	.60 (.34 to .86)		1.30		<.001		.53 (.23 to .84)			1.16		.001	
		Integrated Re-Train vs alternating Re-Train	−.06 (−.32 to .21)		0.12		.68		−.10 (−.40 to .21)			0.21		.53	
**Negative target interpretation (IREC-T)**															
	**Time effect**														
		Integrated Re-Train	−.59 (−.82 to −.36)		1.00		<.001		.01 (−.22 to .24)			0.02		.93	
		Alternating Re-Train	−.64 (−.91 to −.38)		1.10		<.001		−.02 (−.30 to .25)			0.04		.87	
		Control	−.12 (−.34 to .10)		0.20		.29		−.27 (−.50 to −.04)			0.46		.02	
	**Group** × **time interaction**														
		Integrated Re-Train vs control	−.47 (−.79 to −.15)		0.80		.004		.28 (−.05 to .61)			0.48		.09	
		Alternating Re-Train vs control	−.52 (−.87 to −.18)		0.89		.003		.25 (−.11 to .61)			0.42		.18	
		Integrated Re-Train vs alternating Re-Train	.05 (−.29 to .40)		0.09		.76		−.03 (−.33 to .39)			0.06		.86	
**Alcohol approach bias (AAT^c^)**															
	**Time effect**														
		Integrated Re-Train	−33.26 (−93.80 to 27.28)		0.22		.28		10.86 (−56.99 to 78.70)			0.07		.75	
		Alternating Re-Train	−6.45 (−75.71 to 62.80)		0.04		.86		−23.87 (−103.69 to 55.96)			0.16		.56	
		Control	−17.45 (−75.98 to 41.09)		0.12		.56		−57.99 (−124.51 to 8.52)			0.38		.09	
	**Group** × **time interaction**														
		Integrated Re-Train vs control	−15.81 (−100.02 to 68.40)		0.10		.71		68.85 (−26.17 to 163.86)			0.45		.16	
		Alternating Re-Train vs control	10.99 (−79.69 to 101.68)		0.07		.81		34.13 (−69.78 to 138.04)			0.22		.52	
		Integrated Re-Train vs alternating Re-Train	−26.80 (−118.79 to 139.18)		0.18		.57		34.72 (−34.01 to 139.49)			0.23		.52	
**Non–alcohol approach bias (AAT)**															
	**Time effect**														
		Integrated Re-Train	−25.01 (−92.21 to 42.19)		0.12		.47		−10.45 (−91.12 to 70.23)			0.05		.80	
		Alternating Re-Train	36.74 (−40.56 to 114.04)		0.17		.32		54.77 (−40.02 to 149.56)			0.26		.28	
		Control	−30.91 (−95.47 to 33.65)		0.15		.35		−62.62 (−141.49 to 16.25)			0.30		.12	
	**Group** × **time interaction**														
		Integrated Re-Train vs control	5.90 (−87.29 to 99.09)		0.03		.90		52.17 (−60.65 to 165.00)			0.25		.37	
		Alternating Re-Train vs control	67.65 (−33.07 to 168.37)		0.32		.19		117.39 (−5.93 to 240.70)			0.56		.06	
		Integrated Re-Train vs alternating Re-Train	−61.75 (−164.18 to 40.68)		0.29		.24		−65.21 (−189.69 to 59.26)			0.31		.30	
**Comorbid social anxiety and alcohol interpretation bias**															
	**Time effect**														
		Integrated Re-Train	−.68 (−1.05 to −.30)		0.47		<.001		−1.17 (−1.66 to −.68)			0.81		<.001	
		Alternating Re-Train	−.62 (−1.05 to −.18)		0.43		.005		−1.10 (−1.67 to −.52)			0.76		<.001	
		Control	−.40 (−.76 to −.05)		0.28		.02		−.35 (−.81 to .12)			0.24		.14	
	**Group** × **time interaction**														
		Integrated Re-Train vs control	−.27 (−.79 to .24)		0.19		.30		−.83 (−1.50 to −.15)			0.57		.02	
		Alternating Re-Train vs control	−.21 (−.77 to .35)		0.15		.46		−.75 (−1.49 to −.01)			0.52		.048	
		Integrated Re-Train vs alternating Re-Train	−.06 (−.63 to .51)		0.04		.83		−.08 (−.84 to .68)			0.05		.84	

^a^All 3 groups received treatment as usual.

^b^IREC-T: Interpretation Recognition Task.

^c^AAT: Alcohol Approach Task.

**Table 5:** Mixed models for repeated measures fitted to clinical outcomes with time period and intervention group^a^.

Outcomes			Baseline to 6-week follow-up							Baseline to 12-week follow-up				
			β (95% CI)		Cohen *d*		*P* value		β (95% CI)			Cohen *d*		*P* value
**Social anxiety disorder symptoms (SIAS-6^b^ and SPS-6^c^)**														
	**Time effect**													
		Integrated Re-Train	−.46 (−3.32 to 2.41)	0.04		.76		−2.73 (−6.10 to .65)			0.27		.11	
		Alternating Re-Train	−3.01 (−6.30 to .28)	0.29		.07		−4.55 (−8.55 to −.56)			0.44		.03	
		Control	−1.87 (−4.57 to .83)	0.18		.17		−1.00 (−4.22 to 2.22)			0.10		.54	
	**Group** × **time interaction**													
		Integrated Re-Train vs control	1.42 (−2.52 to 5.35)	0.14		.48		−1.73 (−6.39 to 2.94)			0.17		.47	
		Alternating Re-Train vs control	−1.14 (−5.39 to 3.12)	0.11		.60		−3.56 (−8.69 to 1.58)			0.35		.18	
		Integrated Re-Train vs alternating Re-Train	2.55 (−1.81 to 6.91)	0.25		.25		1.83 (−3.40 to 7.06)			0.18		.49	
**Generalized social anxiety (SPWSS^d^)**														
	**Time effect**													
		Integrated Re-Train	−.45 (−.97 to .06)	0.32		.08		−1.01 (−1.60 to −.42)			0.71		.001	
		Alternating Re-Train	−1.01 (−1.60 to −.41)	0.71		.001		−1.89 (−2.59 to −1.18)			1.33		<.001	
		Control	−.57 (−1.06 to −.08)	0.40		.02		−.71 (−1.26 to −.15)			0.50		.01	
	**Group** × **time interaction**													
		Integrated Re-Train vs control	.12 (−.59 to .83)	0.08		.75		−.30 (−1.11 to .51)			0.21		.46	
		Alternating Re-Train vs control	−.43 (−1.20 to .33)	0.31		.27		−1.18 (−2.08 to −.28)			0.83		.01	
		Integrated Re-Train vs alternating Re-Train	.55 (−.24 to 1.34)	0.39		.17		.88 (−.04 to 1.79)			0.62		.06	
**Average number of drinks per day (TLFB)^e,f^**														
	**Time effect**													
		Integrated Re-Train	1.38 (.93 to 2.07)	0.58		.11		1.33 (.80 to 2.22)			0.56		.27	
		Alternating Re-Train	1.08 (.71 to 1.66)	0.45		.71		.83 (.46 to 1.51)			0.60		.55	
		Control	1.19 (.82 to 1.71)	0.49		.36		.92 (.57 to 1.48)			0.38		.73	
	**Group** × **time interaction**													
		Integrated Re-Train vs control	1.16 (.68 to 2.0)	0.49		.57		1.45 (.73 to 2.85)			0.60		.29	
		Alternating Re-Train vs control	.91 (.53 to 1.60)	0.38		.75		.90 (1.21 to 2.39)			0.38		.79	
		Integrated Re-Train vs alternating Re-Train	1.8 (.71 to 2.29)	0.53		.41		1.60 (.74 to 3.44)			0.67		.23	
**Hazardous alcohol use (AUDIT^g^)**														
	**Time effect**													
		Integrated Re-Train	−3.59 (−5.78 to −1.41)	0.57		.001		−6.18 (−9.29 to −3.07)			0.98		<.001	
		Alternating Re-Train	−4.70 (−7.21 to −2.19)	0.74		<.001		−7.29 (−10.96 to −3.63)			1.15		<.001	
		Control	−3.94 (−5.99 to −1.88)	0.62		<.001		−4.52 (−7.47 to −1.57)			0.71		.003	
	**Group** × **time interaction**													
		Integrated Re-Train vs control	.34 (−2.66 to 3.35)	0.05		.82		−1.66 (−5.95 to 2.62)			0.26		.45	
		Alternating Re-Train vs control	−.76 (−4.01 to 2.49)	0.12		.65		−2.78 (−7.48 to 1.92)			0.44		.25	
		Integrated Re-Train vs alternating Re-Train	1.10 (−2.23 to 4.43)	0.17		.52		1.12 (−3.69 to 5.92)			0.18		.65	
**Severity of dependence (SADQ^h^)**														
	**Time effect**													
		Integrated Re-Train	−3.72 (−6.76 to −.69)	0.35		.02		−6.61 (−10.13 to −3.09)			0.62		<.001	
		Alternating Re-Train	−5.58 (−9.07 to −2.08)	0.52		.002		−6.38 (−10.57 to −2.19)			0.60		.003	
		Control	−2.83 (−5.69 to .03)	0.26		.05		−2.07 (−5.42 to 1.27)			0.19		.23	
	**Group** × **time interaction**													
		Integrated Re-Train vs control	−.89 (−5.06 to 3.28)	0.08		.67		−4.54 (−9.39 to .32)			0.42		.07	
		Alternating Re-Train vs control	−2.75 (−7.26 to 1.77)	0.26		.23		−4.31 (−9.66 to 1.05)			0.40		.12	
		Integrated Re-Train vs alternating Re-Train	1.85 (−2.78 to 6.48)	0.17		.43		−.23 (−5.70 to 5.24)			0.02		.93	
**Alcohol craving (ACQ^i^)**														
	**Time effect**													
		Integrated Re-Train	−.62 (−1.06 to −.17)	0.53		.007		−1.27 (−1.81 to −.74)			1.10		<.001	
		Alternating Re-Train	−1.18 (−1.69 to −.67)	1.02		<.001		−1.24 (−1.86 to −.62)			1.07		<.001	
		Control	−.24 (−.67 to .18)	0.21		.26		−.91 (−1.43 to −.39)			0.79		.26	
	**Group** × **time interaction**													
		Integrated Re-Train vs control	−.37 (−.99 to .24)	0.32		.24		−.36 (−1.11 to .38)			0.31		.34	
		Alternating Re-Train vs control	−.94 (−1.60 to −.28)	0.81		.005		−.33 (−1.14 to .48)			0.28		.43	
		Integrated Re-Train vs alternating Re-Train	.57 (−.11 to 1.24)	0.49		.10		−.03 (−0.85 to .78)			0.03		.94	

^a^All 3 groups received treatment as usual.

^b^SIAS-6: 6-item Social Interaction Anxiety Scale.

^c^SPS-6: 6-item Social Phobia Scale.

^d^SPWSS: Social Phobia Weekly Summary Scale.

^e^TLFB: Timeline Follow-back Procedure.

^f^Count data; incidence rate ratios (95% CI) is presented instead of β (95% CI).

^g^AUDIT: Alcohol Use Disorder Identification Test.

^h^SADQ: Severity of Alcohol Dependence Questionnaire.

^i^ACQ: Severity of Alcohol Craving Questionnaire–Short Form–Revised.

#### Cognitive Biases

##### Interpretation Biases

Both *Re-Train Your Brain* groups showed large reductions in interpretation biases from baseline to 6 weeks and moderate to large reductions in interpretation biases from baseline to 12 weeks. No changes were evident over time in the control group. Both intervention groups experienced a greater reduction than the control group in interpretation biases from baseline to 6 weeks, but these improvements did not sustain until 12 weeks. There were large increases in positive interpretations from baseline to 6 weeks, and these gains were sustained until 12 weeks. Positive interpretations increased more in both intervention groups relative to the control group, from baseline to both follow-up time points. For negative interpretations, a large reduction was evident from baseline to 6 weeks for both intervention groups, and a small to moderate reduction was evident from baseline to 12 weeks for the control group. Both intervention groups showed greater reductions in negative interpretations than the control group from baseline to 6 weeks. There were no differences between the *Re-Train Your Brain* groups on interpretation biases.

##### Alcohol Approach Biases

There was no change over time or differences between the groups in change over time with regard to alcohol approach biases or non–alcohol approach biases.

##### Comorbid Interpretation Biases

A small to moderate reduction was apparent across all groups at 6 weeks. For the *Re-Train Your Brain* groups, these reductions remained at 12 weeks, whereas these improvements were not sustained by the control. Both intervention groups experienced reduced comorbidity biases compared with the control group at 12 weeks, with moderate effect sizes. Effects of both intervention groups were comparable over time.

#### Anxiety and Alcohol Use

##### Social Anxiety

The alternating *Re-Train Your Brain* group experienced a small to moderate reduction in social anxiety from baseline to 12 weeks; however, there was no evidence for differences between the groups in change over time. A reduction in generalized social anxiety was observed from baseline to 6 weeks and from baseline to 12 weeks in the alternating *Re-Train Your Brain* and control groups and from baseline to 12 weeks in the integrated group. There was evidence for 1 difference between the groups in change over time, with participants in the alternating group reporting a larger reduction in generalized social anxiety than the control group from baseline to 12 weeks.

##### Alcohol Consumption

There was no statistical evidence for changes over time or differences between the groups in changes over time.

##### Hazardous Alcohol Use

Across all the groups, there was a sustained reduction in hazardous drinking, with the largest reductions at 12 weeks. No changes over time or differences between groups in changes over time were apparent.

##### Alcohol Cravings and Dependence

Alcohol cravings and dependence improved from baseline to 6 weeks and from baseline to 12 weeks in both *Re-Train Your Brain* groups. No changes were observed in the control group. There was evidence for 1 difference between the groups in change over time, with the alternating group reporting significantly greater reductions in cravings than the control group from baseline to 6 weeks (*P*=.005).

### Sensitivity Analysis

Nonnormal distribution of the residuals was observed for anxiety interpretation bias scores, alcohol approach and non–alcohol approach bias scores, social anxiety disorder symptoms, alcohol craving, and severity of dependence. For these variables, the best normality transformation was identified, and analyses were conducted using the transformed data (Multimedia Appendix 1). In all cases, there was no substantive difference in the results using transformed data; thus, results from raw data models are reported for the ease of interpretation.

## Discussion

### Principal Findings

Consistent with the study hypotheses, both *Re-Train Your Brain* program formats were feasible and acceptable for young adults and resulted in greater improvements when coupled with TAU than TAU only in some cognitive and clinical outcomes (ie, anxiety interpretation biases, comorbid social anxiety and alcohol interpretation biases, generalized social anxiety symptoms, and alcohol cravings). Contrary to the hypotheses, the integrated intervention was not associated with greater engagement or clinical effects than the alternating intervention. Only 1 difference was detected between the 2 intervention groups, with the integrated intervention being rated as less simple and user-friendly than the alternating intervention.

### Feasibility and Acceptability

There were strong indicators of *study feasibility*, having exceeded initial recruitment targets (n=90), largely because of the success of social media advertising for this population, as seen in other internet-delivered trials [93]. There were also high levels of consent (99/100, 99%) and few withdrawals (4/100, 4%). Nearly three-quarters (73/100, 73%) of the sample provided complete data for at least 1 of the follow-ups, which is comparable with or higher than what was seen in other web- or app-based IBM or ApBM studies [37-39,41,42,94,95]. Unexpectedly, the follow-up rates at both follow-ups were significantly lower in the alternating intervention group than in the control group. Given that there were no significant differences in follow-up rates between the 2 intervention groups and no baseline characteristics associated with attrition, it is unclear whether particular aspects of the alternating program delivery contributed to this effect.

The data also demonstrated high levels of intervention feasibility with regard to training adherence and attrition. Approximately three-quarters of both the integrated intervention group (27/35, 77%) and alternating intervention group (24/32, 75%) completed the psychoeducational module and at least 1 training session. Moreover, 1 in 3 (25/67, 37%) completed all 10 training sessions, and 1 in 2 (33/67, 49%) completed the optimum dose of 6 training sessions, with approximately 5 training sessions being completed. Rates of training engagement and completion were at the top end of adherence levels reported in similar digital CBM research efficacy studies (eg, with 3%-43% completing all required training sessions [38,42,43,96]) and feasibility studies for digital IBM (eg, 47% in study 2 [97]) and ApBM in clinical settings (eg, 58% [41]). Incorporation of the psychoeducation module, which contained motivational enhancement and a compelling rationale at the outset of the program, may have been particularly important for initial buy-in and setting expectations about the purpose and nature of the intervention. This may have enhanced the motivation to train and thus may have resulted in increased adherence and lower attrition. Future research may consider adopting a similar approach to overcome the high rates of attrition and low adherence reported in prior CBM research (eg, the studies by Wiers et al [38] and Ji et al [43]). However, it should be noted that the adherence rates were lower than those reported in feasibility studies of internet-delivered or app-based IBM programs that are coupled with in-person reminders and motivational enhancement by primary care providers (eg, 75% [98,99]). Future studies may also consider the inclusion of face-to-face support and encouragement by health providers as a way to optimize adherence to adjunctive CBM programs. No serious adverse events were reported, and no adverse events were attributed to either *Re-Train Your Brain* program, suggesting that the interventions can be safely delivered as a supplement to TAU.

Overall, the participants found the program acceptable. Both intervention groups overwhelmingly rated the intervention as credible, user-friendly, and simple. The System Usability Scale scores were equivalent to an “A” grade [67,68], the highest possible rating, indicating that the program is “excellent” and associated with a positive user experience. This is a substantial improvement from previous youth-rated usability scores for *Re-Train Your Brain* (yielded before incorporating feedback from end users and clinicians) [48], and these scores are higher than those of other recent web-based interventions for mental disorders [100-102]. There were also high rates of treatment satisfaction. Scores on the Client Satisfaction Questionnaire among both intervention groups (mean approximately 24) indicate that the participants averaged “mostly satisfied” on each measure item, which is consistent with [97] or higher than [37] ratings provided in previous IBM and ApBM feasibility and acceptability studies. The participants in the integrated group reported finding the program less simple and user-friendly than those in the alternating group, perhaps because of needing to shift focus between the 2 vastly different training tasks within each session. Although quantitative feedback indicated that the program was not particularly enjoyable, motivating, or engaging, open feedback generally commended the program. Contrary to previous literature, which suggests that trial repetition, boredom, and disengagement are serious concerns for CBM training [56,57], qualitative feedback on the aspects of the program they found most and least helpful indicated that the reaction time–based nature of ApBM was “fun” and that IBM had clear implications for real-life application of learnings. This is in line with other recent qualitative studies of app-based ApBM programs [103]. One way to further boost enjoyment and engagement with the program may be to personalize the content of the training such that participants can select personally relevant IBM social scenarios [98] and ApBM alcohol and nonalcohol images [40]. Together, these findings suggest that both modalities of *Re-Train Your Brain* are feasible to deliver in real-world settings and are worthy investments for continued research.

### Preliminary Efficacy

For interpretation biases, there were large reductions over time (at the 6-week and 12-week follow-ups) in both intervention conditions, and large reductions relative to the control at the 6-week follow-up. As part of this, both intervention groups exhibited large increases in positive interpretations of ambiguous information at 6 weeks and 12 weeks, and this was accompanied by large reductions in negative interpretations at 6 weeks, compared with the control group. These findings converge with accumulating evidence on the efficacy of IBM in changing the interpretive style in both adults and young people [23,25,56,104]. For comorbid social anxiety and alcohol interpretation biases, there were a small to moderate reduction in all 3 groups from baseline to the 6-week follow-up and a large reduction in the intervention groups at the 12-week follow-up. Both the alternating and integrated groups showed improved comorbidity interpretation biases compared with the control group at the 12-week follow-up. Changes in interpretation and comorbidity expectancy biases were expected to be accompanied by reduced anxiety symptoms. Such corresponding effects were found for generalized social anxiety, whereby at the 12-week time point, the alternating *Re-Train Your Brain* group demonstrated greater reduction than the control group. The finding that the intervention effects were detectable at the 12-week follow-up is particularly encouraging in light of the brevity (totaling a maximum of 3 h of training), low cost, and simplicity of the intervention. However, there were no differential effects of group on social anxiety symptoms. Future studies with greater statistical power may consider investigating whether changes in these biases mediated changes in clinical symptoms.

Training did not modify alcohol approach biases. The absence of such a training effect may be due to the low reliability of the Approach Avoidance Task [72,105], which is the most widely used measure for alcohol approach biases. Despite this, both intervention groups had moderate to large reductions in the severity of alcohol dependence and craving over the course of the study, with the alternating *Re-Train Your Brain* group experiencing greater reductions in cravings at the 6-week follow-up relative to the control group. A variety of nonspecific factors may have contributed to the observed reduction in cravings, including the devaluation of alcohol, decreased availability and exposure to alcohol due to COVID-19 restrictions, and increased motivation to change. The large, positive reduction in cravings in the alternating group addresses previously reported concerns regarding the safety of exposing patients to alcohol stimuli [106,107]. It also suggests that exposure to alcohol images in 20-minute training blocks via ApBM may reduce the impact that visual alcohol cues have on triggering craving (ie, desensitization), although future research with larger samples is required to confirm these findings.

Although there were no changes in alcohol consumption over time in any group, all 3 groups showed moderate to large reductions in their level of hazardous drinking across the study period (albeit no group differences were detected). The nondifferential finding on alcohol consumption is corroborated by the results of previous web-based ApBM trials, in which reduced drinking was reported across all conditions [38]. This may signify the difficulty of achieving an improvement in drinking outcomes over and above what can be achieved by TAU. Although this study focused on a young treatment-seeking population who reported drinking at hazardous (or greater) levels and were motivated to change their drinking, the effects of ApBM training are reported to be most beneficial for older people with more severe drinking problems (ie, adults with an alcohol use disorder) [30]. It is also suggested that there are likely to be different treatment goals between web-based ApBM studies with problem drinkers (ie, reduced drinking) and in-clinic trials among patients who exhibit alcohol dependence (ie, abstinence) [20,38], which may contribute to the nonexistent or nonspecific effect on some drinking-related outcomes. Another possible explanation for reduced drinking across the 3 conditions is that all the participants completed the 5-minute twice-weekly check-in assessments of their anxiety and alcohol use symptoms, and assessment reactivity (ie, from mere exposure to alcohol questions) may have reduced alcohol use by increasing awareness and problem recognition and motivating behavior change [108,109]. The current sample also had lower initial levels of drinking (average drinks) than youth with anxiety and hazardous drinking in previous studies [93], which may have restricted any potential benefits of training.

### Strengths and Limitations

This study’s strengths include an investigation into the feasibility and acceptability of a comorbidity-focused IBM+ApBM program that is backed by a strong theoretical rationale. By combining IBM and ApBM training, there was potential to enhance the effects on cognitive biases, anxiety, and alcohol outcomes beyond what training with either type of bias individually would provide. The program was delivered via the internet, a preferred treatment modality for young people [49], thus offering participants desired flexibility, anonymity, and convenience. Training was completed at home in naturalistic environments, which may enhance the transfer of training effects to “real-world” experiences. The examination of the 2 different formats of the program was important for furthering our understanding of which generated greater engagement and improvements in clinical outcomes, based on the observed effect sizes. Overall, both formats were equally feasible to deliver and accepted by young people, with the exception of the simplicity of use, which was rated lower in the integrated group. The follow-up rates were also lower in the alternating group than in the control group. With regard to efficacy, the alternating group showed larger effect sizes over time than the integrated group and had greater impacts on cognitive and clinical outcomes than the control group. The alternating delivery model may be a preferred option for future comorbid anxiety-alcohol CBM research, albeit efforts should be made to improve follow-up rates in this model. Alternatively, the format of delivery might be decided based on an individual user’s preference for an integrated or alternating format, rather than assuming a one-size-fits-all approach to selecting the optimal delivery format. Future research may benefit from developing simple protocols for establishing within-person preferences regarding the 2 delivery formats. The study also had relatively low treatment and study attrition.

The trial also had several limitations, primarily its small pilot trial sample size and use of a relatively homogeneous, nondiagnosed sample. Although the study had an appropriate size for examining feasibility and acceptability, it did not have adequate power to properly test its efficacy in improving cognitive biases or clinical symptoms, which may have biased the estimated effect sizes. The study also included multiple secondary outcome measures for efficacy without an adjustment of the *P* value, which increases the risk of type 1errors (ie, findings of false “significance”). However, because of the pilot nature of the study, which is not powered for efficacy analyses, the results focus on the magnitude of effect rather than on statistical significance. Another limitation is that given that TAU alone can have powerful clinical effects (particularly when treatment is standardized), a substantially larger sample could be required to detect benefits beyond those conferred by TAU. Future trials with a larger sample size could examine this further and determine whether a change in biases mediates changes in clinical symptoms as well as whether the effects are moderated by age, sex, and TAU type and stage. Another limitation was that the control condition was not matched to the *Re-Train Your Brain* program. The selection of a suitable or optimal control condition is a commonly reported challenge in past CBM research, especially given that sham training (a “neutral” control condition) may actually function as an “active” condition rather than the neutral condition it is intended to be [110]. The *Re-Train Your Brain* interventions are targeted at individuals with co-occurring social anxiety and hazardous alcohol use, yet few participants in this trial reported social anxiety as their primary concern. Although this has the potential to influence the generalizability of the findings, two-thirds to three-quarters of the participants across the conditions met the criteria for possible social anxiety disorder; therefore, generalizability should be relatively unaffected.

A further limitation of this study was that although hazardous use in the past year was an eligibility criterion, 1 in 3 participants had not consumed alcohol in the past month at baseline. This was largely observed among the participants in the integrated intervention group (a chance finding, where over half of the group had not consumed any alcohol in the month before baseline). It is unclear whether this low level of use may have been a by-product of the sample receiving psychological treatment or whether there were potential impacts of COVID-19 social isolation rules and restrictions. Future studies could consider incorporating an eligibility criterion that ensures that participants are *current* hazardous drinkers or, if statistical power allows, run subgroup analyses among current drinkers to better understand the intervention effects among the intended target population of current hazardous drinkers.

In addition, specific feasibility benchmarks were unfortunately not prespecified, and the study was unable to investigate the efficacy of the intervention as a function of the type of TAU the participants were receiving. TAU is typically standardized across groups in CBM research (eg, ApBM plus residential inpatient alcohol treatment) [29-33] to prevent variability in the intensiveness and effectiveness of TAU and any potential masking of CBM intervention effects. Although this study did not standardize TAU, we would argue that this approach is still ecologically valid in terms of evaluating whether it would add to routine care. It was also assumed that any heterogeneous external treatment effects would be minimized in this study because of randomization to conditions. Future research with a larger sample may consider examining whether *Re-Train Your Brain’s* efficacy is greater when used as a supplement to particular types of treatment.

### Conclusions

For the first time, this study demonstrated the feasibility and acceptability of delivering a comorbidity-focused, web-based IBM plus ApBM program as an adjunct treatment for youth with co-occurring social anxiety and hazardous drinking. Both formats of the *Re-Train Your Brain* intervention were associated with improvements in cognitive biases, and the alternating intervention had additional benefits on some anxiety- and drinking-related outcomes relative to the control, although other clinical outcomes suggested comparable changes for the TAU control condition. Internet-delivered IBM+ApBM training offered through a publicly available website may represent a scalable, low-cost, and non–labor-intensive intervention for targeting interpretation, alcohol approach, and comorbidity biases as well as anxiety symptom severity and alcohol-related outcomes in the real world. Further research (eg, fully powered RCTs) into this adjunct treatment approach is clearly warranted.

## Appendix

Multimedia Appendix 1 Supplementary materials containing full description of study measures and the sensitivity analyses.

## Abbreviations

ApBM: approach bias modification
AUDIT: Alcohol Use Disorder Identification Test
CBM: cognitive bias modification
CONSORT: Consolidated Standards of Reporting Trials
IBM: interpretation bias modification
OR: odds ratio
RCT: randomized controlled trial
SPIRIT: Standard Protocol Items: Recommendations for Interventional Trials
TAU: treatment as usual
