# Supplementary material for: Web-Based Cognitive Bias Modification Program for Young People With Social Anxiety and Hazardous Alcohol Use: Feasibility, Acceptability, and Preliminary Efficacy Study
Source: JMIR Form Res. 2023 Oct 25;7:e46008. doi: 10.2196/46008 (PMC10632924; doi:10.2196/46008)
Supplement: Multimedia Appendix 1 [file formative_v7i1e46008_app1.docx]

# Measures

## Feasibility

*Feasibility of the research* study was measured by the proportion of participants who provided consent, completed follow-up assessments, and withdrew from the study. *Feasibility of the intervention* was measured by the proportion of participants who commenced training, completed all 10 training sessions, completed the optimum number of six training sessions for efficacy effects, based on past ApBM research (i.e., the number of sessions before the point where the learning curve becomes flat)[65], and the number of sessions completed. Feasibility was also assessed by the reporting of adverse events via spontaneous reports to the research team or open feedback questions in the 6-week survey.

## Acceptability

*Usability* of the program was assessed among the two intervention groups at 6-weeks post-baseline using the System Usability Scale[66](Cronbach’s α=.794), where scores range from 0 to 100, with higher scores indicating greater usability. A score greater than 68 is considered above average[67]. Cut-off scores using a curved grading scale were used to interpret scores (0–51.6=F, 51.7–62.6=D, 62.7–72.5=C, 72.6–78.8=B, 78.9–100=A)[67], and described using adjective ratings[68]. *Satisfaction* was measured by the Client Satisfaction Questionnaire-8[69](Cronbach’s α=.953), where scores range from 8 to 32, and a higher score denotes greater client satisfaction. Acceptability was also assessed by 13 acceptability items and several open-ended questions (developed by the research team) asking which features and aspects of the program they found most/least helpful. To determine which intervention delivery model was preferred, participants were asked four *user experience* items, including whether they felt motivated for training, whether training was enjoyable, whether they liked the delivery of training, and whether the program was simple and easy to use. All items were rated on a 5-point Likert scale from 0 ‘Not at all’ to 4 ‘Extremely’. For analyses, binary variables were created to represent 0 ‘Not at all/a little/somewhat' and 1 'Very/extremely'.

## Interpretation and Alcohol Approach Biases

*Social anxiety interpretation biases* were measured by the Interpretation Recognition Task[70,71], which contains two components: an encoding phase and a recognition test phase. During the encoding phase, participants were asked to read a set of 10 ambiguous scenarios presented on a computer screen; each scenario comprised a title and three sentences that were ambiguous in terms of valence. In the final sentence, a word fragment was presented. After reading each scenario, participants completed the word fragment as quickly as possible by pressing the spacebar when they knew what the word was (reaction time was recorded), followed by the key corresponding to the missing letter. Participants then answered “yes” or “no” to a fact-based comprehension question about the scenario, which was followed by feedback (i.e., “correct” or “incorrect”). Neither the word fragment nor the comprehension question disambiguated the scenario. An example of the encoding phase is provided below:

(Title): *The evening class.*

(Scenario): *You’ve just started going to an evening class. The instructor asks a question and no one in the group volunteers an answer, so he looks directly at you. You answer the question, aware of how your voice must sound to the ….*

(Word fragment): *oth-rs*

(Correct word): *others*

(Comprehension question): *Have you been going to the evening class for a long time?*

(Response): *No*

(Feedback): ✓ *Correct answer*

After reading and imagining themselves in each of the scenarios, as part of the recognition test phase, participants were presented with the title of each scenario, followed by four disambiguated interpretations of each scenario, presented one at a time. Participants rated each interpretation for its similarity in meaning to the original scenario on a 4-point Likert scale from 1 ‘very different in meaning’ to 4 ‘very similar in meaning’. The four interpretations contained one valid, positively valanced interpretation (positive target e.g., “You answer the question, aware of the others listening attentively”), one valid, negative valanced interpretation (negative target e.g., “You answer the question, aware of how unsteady your voice sounds”), one positively valanced statement that was unrelated to the potential threat of the original scenario and did not reflect anxiety-linked concerns (positive foil e.g., “You answer the question and then realise what a good answer it is”), and one negatively valanced statement that was unrelated to the potential threat of the original scenario and did not reflect anxiety-linked concerns (negative foil e.g., “You answer the question but realise that you have made a mistake”). Mean recognition ratings were individually calculated so that average scores (ranging from 1 to 4) for each of the four interpretations were generated. Mean ratings of positive (Cronbach’s α = .714) and negative (Cronbach’s α = .824) target interpretations were the dependent measures. To create an interpretation bias score, mean similarity scores of positive interpretations were subtracted from mean similarity scores from negative interpretations, with higher scores indicating a stronger threat-related interpretive bias. A score above zero on the interpretation bias score was used to indicate an interpretation bias.

*Alcohol approach biases* were assessed using the Alcohol Approach Task[15], where participants were instructed to pull or push a computer mouse based on whether images shown on a computer screen were in portrait or landscape orientation. Twenty images of alcoholic beverage and 20 colour- and shape-matched images of non-alcoholic beverages were used. Contingent upon a pull or push movement, the picture increased or decreased in size to represent an approach or avoidance behaviour. Each image was repeated twice, for a total of 80 trials. Images were selected based on the beverage type and brands commonly used by the target age group[48]. Format movement assignments (i.e., push or pull portrait or landscape images) were counterbalanced and feedback was provided for correct (green ‘✓’) and incorrect responses (red ‘X’). Participants were required to correct any incorrect responses in order to proceed. Ten practice trials with empty rectangular frames were used. As per previous studies[32,72], trials were considered valid if the initial push/pull response was correct and the reaction time was 300-3000ms. Median reaction times were calculated separately for alcohol-pull, alcohol-push, non-alcohol-pull, non-alcohol-push responses. Approach bias was calculated separately for each picture type (alcohol; non-alcohol) by subtracting the median reaction time for pull responses from the median reaction time for push responses. A positive score was indicative of an approach tendency to relevant stimuli. To provide an index of alcohol approach bias relative to non-alcohol-related bias, the non-alcohol approach bias scores were then subtracted from the alcohol approach bias scores.

*Interpretation biases for co-occurring social anxiety and alcohol use* were assessed using the Comorbid Social Anxiety and Alcohol Interpretation Bias task[73]. Participants were presented with a set of eight ambiguous social scenarios related to: drinking to manage a social situation, drinking to reduce shame after a social situation, and embarrassment after a heavy drinking episode. Each scenario was followed by three possible explanations for the situation. One of the explanations for each scenario was associated with a link between social concerns and alcohol use (e.g., “You want a drink so you will have an easier time talking to people”), whereas the other responses were not (e.g., *“*You are thirsty and want to grab a soda.”). Participants were asked to rate the degree to which each of the three explanations would likely be true if they were in that situation (0 ‘not at all likely’ to 8 ‘extremely’). The item score for the comorbidity-relevant response option was averaged across the set of scenarios and divided by the average score across all other item responses. For descriptive purposes, raw means scores across the comorbidity-relevant response options are provided, with higher scores indicating a stronger comorbidity bias[73].

## Anxiety

*Social anxiety disorder symptoms* were assessed using the 12-item Social Interaction Anxiety Scale (SIAS-6) and Social Phobia Scale-short forms (SPS-6)[62]. The SIAS-6 and SPS-6 assess the degree to which 12 common social interaction anxieties (e.g., “I have difficulty talking with other people”) and performance anxieties (e.g., “I worry about shaking or trembling when I’m watched by other people”) are characteristic of the respondent. There is no particular timeframe for these questions. Items are scored on a 5-point Likert scale, ranging from 0 being ‘not at all true or characteristic of me’ to 4 being ‘extremely true or characteristic of me’. Cut-off scores of ≥7 on the SIAS-6 and ≥2 on the SPS-6 are indicative of a possible diagnosis of social phobia (used for inclusion in current study). A total composite score was created to assess the degree of social anxiety symptomology across social interaction and performance-based anxieties, with scores ranging from 0 to 48. Higher scores indicated greater social anxiety. *Generalised social phobia* was assessed using the 6-item Social Phobia Weekly Summary Scale[74]. The measure assessed the severity and frequency of social anxiety, social avoidance, self-focused versus external attention (in general and in difficult social situations)*,* anticipatory processing, and post-event rumination over the past week. Each item was rated on a Likert-type scale ranging from 0 to 8, and higher scores denoted greater social anxiety symptomology.

## Alcohol use

*Average drinks per day* in the past month were assessed via a computerised version of the Timeline Follow-back Procedure[75-77]. *Hazardous alcohol use* was assessed through the 10-item AUDIT[61]. Total scores range from 0–40, and cut-off scores of 8–15, 16–25, and ≥26 were used to indicate risky/hazardous alcohol use, high risk/harmful use, and high risk/possible dependency. *Severity of alcohol dependence* was assessed by the 20-item Severity of Alcohol Dependence Questionnaire[78]. Each item was scored from 0 ‘almost never’ to 3 ‘nearly always’, with total scores ranging from 0 to 60. Higher scores indicated greater dependence on alcohol, with the following cut-offs used to indicate non-dependence (score 0 – 7), mild, dependence (8 – 15), moderate dependence (16 – 30), and severe dependence (31 - 60). *Alcohol cravings* were assessed by the 12-item Severity of Alcohol Craving Questionnaire–Short Form–Revised[79]. Each item was scored from 1 ‘strongly disagree’ to 7 ‘strongly agree’, and higher scores indicated stronger cravings for alcohol.

## Additional variables

Participants were asked about their *sociodemographic characteristics* including age, sex, education, employment, country of birth, and primary mental health/substance use concern. *Frequency of other drug use* including cannabis, non-prescribed benzodiazepines, and psychostimulants in the past year was assessed by the National Institute on Drug Abuse quick screen[81]. *Readiness and motivation to change* anxiety and/or alcohol use were assessed via a readiness ruler (e.g., on a scale of 1 to 10, how ready are you to change your anxiety/drinking), as well as using the University of Rhode Island Change Assessment[80]. Cut-off scores on the University of Rhode Island Change are used to describe four stages of change: (1) precontemplation (score 0-8), where an individual has no desire and is not intending to make any changes; (2) contemplation (score 8-11), where an individual is considering how they may go about making change; (3) action/preparation (score 11-14), where an individual has started making changes; and (4) maintenance (score 14+), where an individual has already made changes and wishes to maintain them. *Psychological and pharmacological treatment* received in the past 3 months included: i) whether participants had received any treatment from a health professional for anxiety symptoms or alcohol use problems in the past 3 months (yes/no), ii) who they consulted, iii) the type of professional they consulted i.e., counsellor, psychologist, clinical psychologist, GP, psychiatrist, hospital inpatient admissions, inpatient or residential treatment, emergency department visits, other, iv) what they consulted them for i.e., anxiety, drinking, other, v) whether treatment was current (yes/no), and vi) what type of treatment they received i.e., cognitive behavioural therapy, counselling, alcohol/other drug counselling, dialectical behavioural therapy, acceptance & commitment therapy, motivational interviewing, schema therapy, other, or unsure. Participants were asked whether they had taken any medication for anxiety, depression, or alcohol use problems, what the medication was, when they started/ceased taking them, and whether medications were current (yes/no).

# Interventions

## IBM for social anxiety

In each session, participants were provided a set of ambiguous social scenarios consisting of three lines that were presented one-by-one on a computer screen by pressing the spacebar. The final line of each scenario contained a word fragment, and participants were instructed to press the spacebar as soon as they knew what the word was (reaction times were recorded). They then filled in the missing letter using their keyboard (the program only continued once a correct response was provided). The word fragment always resolved the ambiguity in a positive or neutral way. After each scenario, participants responded to a yes/no comprehension question and received feedback (“correct” or “incorrect”, with a corresponding smiley or sad face emoji). To ‘gamify’ the task, points were awarded for each correct letter (+1) and comprehension question response (+1). The scenarios were generated specifically for Australian youth[48]. Three practice trials were given prior to the real training.

## ApBM for alcohol use

Participants were instructed to pull or push a computer mouse in response to the orientation of images containing alcoholic or non-alcoholic beverages (e.g., pull landscape and push portrait), and the picture size increased or decreased accordingly. Ninety-five percent of image orientations used to train avoidance behaviours (i.e., a push movement) contained images of alcoholic beverages, while the remaining 5% contained images of non-alcoholic beverages (and vice versa for orientations used to train approach behaviours). Format movements were counterbalanced so that half of the participants pulled landscape and pushed portrait images, while remaining participants received the opposite instruction. Participants received ‘gamified’ feedback for correct (green ‘✓’ plus smiley emoji) and incorrect responses (red ‘X’ plus sad emoji). Images were repeated for incorrect movements while points were awarded for each correct movement (+1) to enhance personal engagement and motivation. Participants were given 5 practice trials (i.e., empty rectangular frames). The alcoholic and non-alcoholic images were based on beverage types and brands commonly consumed by young people[48].

# Results

**Sensitivity analysis: Efficacy outcomes using the best normality transformation**

| Table S1. Mixed models for repeated measures fitted to cognitive bias outcomes with time period and intervention group | | | | | | | |
| --- | --- | --- | --- | --- | --- | --- | --- |
|  | | Baseline to 6-week follow-up | | | Baseline to 3-month follow-up | | |
| Outcomes | | *β* [95% CI] | *Cohen’s d* | *p-value* | *β* [95% CI] | *Cohen’s d* | *p-value* |
| Anxiety interpretation bias (IREC-T) ^a^ | | |  |  |  |  |  |
| Time effect | | |  |  |  |  |  |
|  | Control | 0.05 [-0.33, 0.44] | 0.05 | .796 | -0.36 [-0.80, 0.08] | 0.36 | .112 |
|  | *Re-Train* *Integrated* | **-1.34** [-1.73, -0.94] | 1.34 | <.001 | **-0.65** [-1.09, -0.20] | 0.65 | .005 |
|  | *Re-Train* *Alternating* | **-1.48** [-1.94, -1.03] | 1.49 | <.001 | **-0.73** [-1.25, 0.21] | 0.73 | .006 |
| Group x Time Interaction | | | | | | | |
|  | *Re-Train Integrated* v Control | **-1.39** [-1.94, -0.83] | 1.39 | <.001 | -0.29 [-0.92, 0.34] | 0.29 | .375 |
|  | *Re-Train Alternating* v Control | **-1.53** [-2.13, -0.94] | 1.54 | <.001 | -0.37 [-1.06, 0.31] | 0.37 | .287 |
|  | *Re-Train Integrated* v *Alternating* | 0.15 [-0.46, 0.75] | 0.15 | .634 | 0.09 [-0.60, 0.77] | 0.09 | .804 |
| Alcohol approach bias (AAT) ^a^ | | |  |  |  |  |  |
| Time effect | | |  |  |  |  |  |
|  | Control | -0.07 [-0.50, 0.37] | 0.07 | -760 | -038 [-0.85, 0.10] | 0.38 | .123 |
|  | *Re-Train* *Integrated* | -0.29 [-0.73, 0.16] | 0.29 | -214 | -0.07 [-0.41, 0.56] | 0.07 | .769 |
|  | *Re-Train* *Alternating* | -.04 [-0.55, 0.48] | 0.03 | .896 | -0.15 [-0.73, 0.42] | 0.15 | .599 |
| Group x Time Interaction | | | | | | | |
|  | *Re-Train Integrated* v Control | -0.22 [-0.84, 0.41] | 0.22 | .497 | 0.45 [-0.23, 1.13] | 0.45 | .197 |
|  | *Re-Train Alternating* v Control | -0.03 [-0.64, 0.71] | 0.03 | .922 | 0.22 [-0.53, 0.97] | 0.22 | .560 |
|  | *Re-Train Integrated* v *Alternating* | -0.25 [-0.93, 0.43] | 0.25 | .471 | 0.23 [-0.53, 0.98] | 0.23 | .555 |
| Non-alcohol approach bias (AAT) ^a^ | | |  |  |  |  |  |
| Time effect | | |  |  |  |  |  |
|  | Control | -0.18 [-0.59, 0.23] | 0.18 | .395 | -0.37 [-0.86, 0.11] | 0.37 | .133 |
|  | *Re-Train* *Integrated* | -0.12 [-0.55, 0.30] | 0.12 | .568 | -0.01 [-0.51, 0.48] | 0.01 | .965 |
|  | *Re-Train* *Alternating* | 0.22 [-.26, 0.71] | 0.22 | .370 | -0.28 [-0.30, 0.86] | 0.28 | .351 |
| Group x Time Interaction | | | | | | | |
|  | *Re-Train Integrated* v Control | 0.05 [-0.53, 0.64] | 0.05 | .857 | 0.36 [-0.33, 1.05] | 0.36 | .308 |
|  | *Re-Train Alternating* v Control | 0.40 [-0.23, 1.03] | 0.40 | .217 | 0.65 [-0.11, 1.40] | 0.65 | .093 |
|  | *Re-Train Integrated* v *Alternating* | -0.34 [-0.99, 0.30] | 0.35 | .294 | -0.29 [-1.05, 0.48] | 0.29 | .461 |
| ^a^ Inverse rank transformed data | | | | | | | |
| Table S2. Mixed models for repeated measures fitted to clinical outcomes with time period and intervention group | | | | | | | |
|  | | Baseline to 6-week follow-up | | | Baseline to 3-month follow-up | | |
| Outcomes | | *β* [95% CI] | *Cohen’s d* | *p-value* | *β* [95% CI] | *Cohen’s d* | *p-value* |
| Social anxiety disorder symptoms (SIAS-6 & SPS-6) ^a^ | | |  |  |  |  |  |
| Time effect | | |  |  |  |  |  |
|  | Control | -0.21 [-0.52, 0.10] | 0.17 | .190 | -0.06 [-0.44, 0.31] | 0.05 | .736 |
|  | *Re-Train* *Integrated* | -0.11 [-0.44, 0.22] | 0.09 | .523 | -0.36 [-0.75, 0.03] | 0.30 | .071 |
|  | *Re-Train* *Alternating* | -0.32 [-0.69, 0.06] | 0.27 | .102 | -0.41 [-0.87, 0.05] | 0.34 | .083 |
| Group x Time Interaction | |  |  |  |  |  |  |
|  | *Re-Train Integrated* v Control | 0.10 [-0.35, 0.55] | 0.08 | .665 | -0.30 [-0.83, 0.24] | 0.25 | .282 |
|  | *Re-Train Alternating* v Control | -0.11 [-0.60, 0.38] | 0.09 | .663 | -0.34 [-0.94, 0.25] | 0.29 | -254 |
|  | *Re-Train Integrated* v *Alternating* | 0.21 [-0.29, 0.71] | 0.18 | .414 | 0.05 [-0.55, 0.65] | 0.04 | .873 |
| Severity of dependence (SADQ) ^a^ | | |  |  |  |  |  |
| Time effect | | |  |  |  |  |  |
|  | Control | **-0.53** [-0.96, -0.10] | 0.41 | .015 | -0.37 [-0.85, 0.11] | 0.29 | .130 |
|  | *Re-Train* *Integrated* | **-0.58** [-1.04, -0.13] | 0.45 | .011 | **-0.95** [-1.45, -0.44] | 0.73 | <.001 |
|  | *Re-Train* *Alternating* | **-0.87** [-1.39, -0.35] | 0.69 | .001 | **-0.96** [-1.57, -0.35] | 0.74 | .002 |
| Group x Time Interaction | | | | | | | |
|  | *Re-Train Integrated* v Control | -0.05 [-0.68, 0.57] | 0.04 | .864 | -0.58 [-0.13, 0.12] | 0.44 | .107 |
|  | *Re-Train Alternating* v Control | -0.34 [-1.01, 0.34] | 0.26 | .326 | -0.59 [-1.37,0.18] | 0.46 | .134 |
|  | *Re-Train Integrated* v *Alternating* | 0.28 [-0.41, 0.97] | 0.22 | .421 | 0.02 [-0.78, 0.81] | 0.01 | .964 |
| Alcohol craving (ACQ) ^a^ | | |  |  |  |  |  |
| Time effect | | |  |  |  |  |  |
|  | Control | -0.08 [-0.20, 0.04] | 0.25 | .201 | **-0.26** [-0.40, -0.11] | 0.81 | <.001 |
|  | *Re-Train* *Integrated* | **-0.18** [-0.30, -0.05] | 0.57 | .005 | **-0.39** [-0.54, -0.24] | 1.23 | <.001 |
|  | *Re-Train* *Alternating* | **-0.37** [-0.51, -0.23] | 1.17 | <.001 | **-0.34** [-0.52, -0.17] | 1.09 | <.001 |
| Group x Time Interaction | | | | | | | |
|  | *Re-Train Integrated* v Control | -0.10 [-0.27, 0.07] | 0.32 | .246 | -0.13 [-0.34, 0.08] | 0.41 | .225 |
|  | *Re-Train Alternating* v Control | **-0.29** [ -0.48, -0.11] | 0.93 | .002 | -0.29 [-0.48, 0.14] | 0.30 | .452 |
|  | *Re-Train Integrated* v *Alternating* | 0.19 [0.00, 0.38] | 0.60 | .051 | -0.04 [-0.27, 0.19] | 0.14 | .719 |
| ^a^ Square root transformed data | | | | | | | |
